# Caveolin-1 inhibits breast cancer stem cells via c-Myc-mediated metabolic reprogramming

**DOI:** 10.1038/s41419-020-2667-x

**Published:** 2020-06-11

**Authors:** Shengqi Wang, Neng Wang, Yifeng Zheng, Bowen Yang, Pengxi Liu, Fengxue Zhang, Min Li, Juxian Song, Xu Chang, Zhiyu Wang

**Affiliations:** 1 Integrative Research Laboratory of Breast Cancer, the Research Center for Integrative Cancer Medicine, Discipline of Integrated Chinese and Western Medicine & the Second Clinical College of Guangzhou University of Chinese Medicine, Guangzhou, 510006 China; 2 Guangdong Provincial Key Laboratory of Clinical Research on Traditional Chinese Medicine Syndrome, Guangdong Provincial Academy of Chinese Medical Sciences, Guangdong Provincial Hospital of Chinese Medicine, Guangzhou, 510006 China; 3 Post-doctoral Research Center, Guangzhou University of Chinese Medicine, Guangzhou, 510006 China; 4 College of Basic Medicine, Guangzhou University of Chinese Medicine, Guangzhou, 510006 China; 5 School of Chinese Medicine, Hong Kong Baptist University, 999077 Kowloon Tong, Hong Kong; 6 Department of Mammary Disease, Panyu Hospital of Chinese Medicine, Guangzhou, 511400 China

**Keywords:** Breast cancer, Cancer stem cells

## Abstract

Breast cancer stem cells (BCSCs) are considered to be the root of breast cancer occurrence and progression. However, the characteristics and regulatory mechanisms of BCSCs metabolism have been poorly revealed, which hinders the development of metabolism-targeted treatment strategies for BCSCs elimination. Herein, we demonstrated that the downregulation of Caveolin-1 (Cav-1) usually occurred in BCSCs and was associated with a metabolic switch from mitochondrial respiration to aerobic glycolysis. Meanwhile, Cav-1 could inhibit the self-renewal capacity and aerobic glycolysis activity of BCSCs. Furthermore, Cav-1 loss was associated with accelerated mammary-ductal hyperplasia and mammary-tumor formation in transgenic mice, which was accompanied by enrichment and enhanced aerobic glycolysis activity of BCSCs. Mechanistically, Cav-1 could promote Von Hippel-Lindau (VHL)-mediated ubiquitination and degradation of c-Myc in BCSCs through the proteasome pathway. Notably, epithelial Cav-1 expression significantly correlated with a better overall survival and delayed onset age of breast cancer patients. Together, our work uncovers the characteristics and regulatory mechanisms of BCSCs metabolism and highlights Cav-1-targeted treatments as a promising strategy for BCSCs elimination.

## Introduction

Breast cancer is the most commonly diagnosed malignancy (24.2% of new cancer diagnoses) and the leading cause of cancer-related deaths (15.0%) among women worldwide^1–3^. The prevalence of breast cancer is still increasing globally every year, especially in young women^2^. Thus, it is of great urgency and clinical significance to identify novel antitumor strategies and targets for the prevention and treatment of breast cancer.

Metabolic reprogramming is considered as one of the hallmarks of cancer^4^. Otto Warburg firstly observed that glucose uptake and lactate production were dramatically increased in cancer cells even in the presence of oxygen, which has been termed aerobic glycolysis^5^. Considering that aerobic glycolysis is an essential characteristic of aggressive cancer cells that differs from normal cells^4^, targeting aberrant glycolytic metabolism may be a promising strategy for cancer treatment. Multiple glycolytic inhibitors including 2-deoxyglucose (2-DG) and 3-bromopyruvate (3-BrPA) have shown excellent antitumor activities in breast cancer xenografts^6^. Cancer stem cells (CSCs) are at the root of cancer drug resistance and metastasis. Notably, several reports have suggested that CSCs prefer a glycolytic phenotype^7,8^. Targeting the metabolism of CSCs may be a promising strategy for cancer treatment. Therefore, it is of considerable value to explore the key targets involved in aerobic glycolysis modulation in CSCs. However, the existing knowledge about the characteristics and regulatory mechanisms of CSCs metabolism is poorly understood.

Caveolin-1 (Cav-1) is a constituent protein of flask-shaped plasma membrane invaginations called caveolae. Multiple studies reported that stromal Cav-1 loss predicted a poor overall survival or cancer progression in breast cancer patients^9–11^, while epithelial Cav-1 was marginally significant^11^. With no discrimination between epithelial and stromal Cav-1, Hart et al. also reported that low Cav-1 expression in invasive ductal carcinoma was associated with lower five-year survival rates and molecular subtypes with the poorest prognosis^12^. In terms of mechanisms, stromal Cav-1 loss could promote tumor progression through various paracrine signaling mechanisms including extracellular matrix remodeling, fibrosis, and modulation of the tumor microenvironment^13^. Meanwhile, Cav-1 has also been implicated in cancer metabolic modulation^14^. For example, Cav-1 reconstitution could suppress aerobic glycolysis in breast cancer cells^12^. However, it is unknown whether Cav-1 loss contributes to the aerobic glycolysis phenotype of Breast cancer stem cells (BCSCs) or not.

Recently, an increasing interest has been devoted to exploring the possible correlation between Cav-1 and stem cells. Sotgia et al. reported that Cav-1 inactivation could induce an increased stem signaling of Wnt/β-catenin during mammary tumorigenesis^15^. Additionally, Cav-1 knockdown could promote the proliferation of human mesenchymal stem cells^16^. With regard to stem cell differentiation, the latest research indicates that Cav-1 is essential in the differentiation of human adipose-derived stem cells into hepatocyte-like cells^17^. Mechanistic studies suggest that Cav-1 expression and caveolae structure could help maintain pluripotency marker expression in mouse embryonic stem cells^18^. All these findings suggest that Cav-1 might influence the self-renewal and multi-differentiation processes of stem cells. Although several reports have indicated the enrichment of Cav-1 expression in CSCs^19,20^, its relative level when normalized to that of normal stem cells and its biological function in regulating the metabolism of CSCs are still unclear and worth for further investigations.

Herein, we systematically investigated the influence of epithelial Cav-1 on the metabolic regulation of BCSCs and the underlying mechanisms.

## Results

### Cav-1 is critical for aerobic glycolysis modulation of breast cancer cells in vitro

**Fig. 1:**
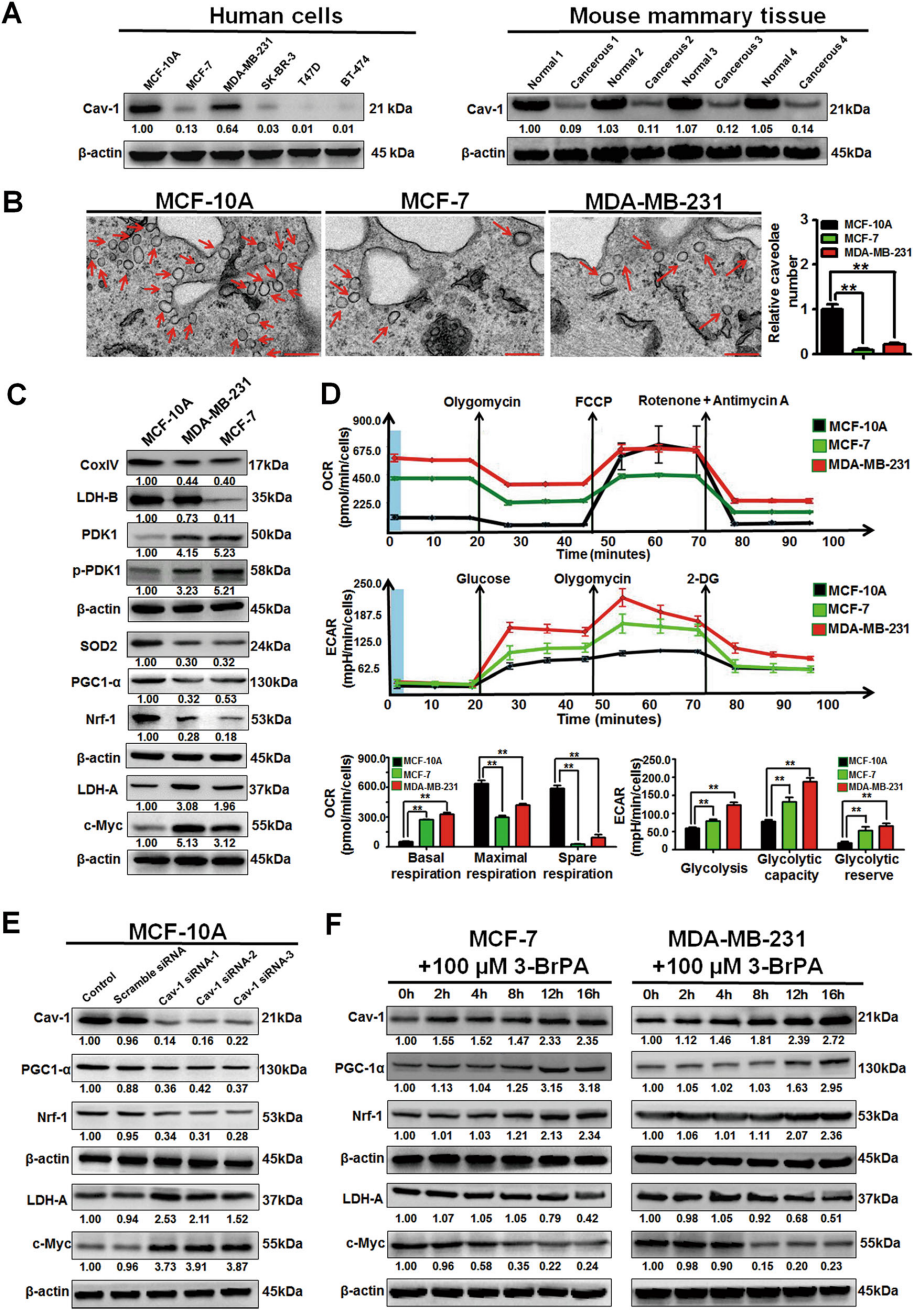
Breast cancer cells exhibit decreased expression of Cav-1 and reprogrammed metabolic patterns that shift from mitochondrial respiration to aerobic glycolysis. **a** Five kinds of human breast cancer cells exhibited decreased expression levels of Cav-1 when compared with that of the nonmalignant mammary epithelial cell line MCF-10A. Meanwhile, Cav-1 expression levels also decreased in mammary-tumor tissues obtained from transgenic MMTV-Wnt1 mice when compared with those of normal mammary tissues obtained from wild type mice. *n* = 3. **b** Less caveolae were organized in MDA-MB-231 and MCF-7 cells when compared with those in MCF-10A cells. Scale bar = 200 nm, *n* = 3. **c** The expression levels of mitochondrial respiration-associated proteins (Nrf-1, PGC1-α, LDH-B, SOD2, and CoxIV) in both MDA-MB-231 and MCF-7 cells were downregulated whereas the expression levels of glycolysis-related proteins (c-Myc, LDH-A, PDK1, and p-PDK1) were upregulated when compared with those in MCF-10A cells. *n* = 3. **d** The Seahorse XF24 extracellular flux analyzer was used to detect OCRs to measure mitochondrial respiration, and the ECARs to determine glycolysis. MCF-7 and MDA-MB-231 cells displayed impaired mitochondrial respiration as well as elevated glycolytic activity when compared with those in MCF-10A cells. *n* = 3. **e** Cav-1-specific siRNAs elevated expression levels of c-Myc and LDH-A while attenuating expression levels of Cav-1, Nrf-1, and PGC1-α in MCF-10A cells. *n* = 3. **f** The time course of the indicated target proteins responses upon 3-BrPA treatment (100 μM). *n* = 3. All values are presented as the mean ± SD, **P < 0.01.

Compared with the nonmalignant mammary epithelial cell line MCF-10A, Cav-1 expression was significantly decreased in multiple human breast cancer cell lines. Similarly, mouse mammary-tumor tissues also exhibited reduced Cav-1 expression when compared with that of normal mammary tissues (Fig. 1a). Cav-1 is the major structural protein of caveolae. In situ observations also indicated that fewer caveolae were organized in MDA-MB-231 and MCF-7 cells when compared with that of MCF-10A cells (Fig. 1b). Furthermore, compared with MCF-10A cells, the expression levels of mitochondrial respiration-associated proteins in MDA-MB-231 and MCF-7 cells were downregulated, whereas the expression levels of glycolysis-related proteins were upregulated (Fig. 1c). More importantly, MCF-7 and MDA-MB-231 cells displayed impaired mitochondrial respiration and elevated glycolytic activity (Fig. 1d). Altogether, breast cancer cells exhibit decreased expression of Cav-1, as well as reprogrammed metabolic patterns, shifted from mitochondrial respiration to aerobic glycolysis.

Next, to investigate the correlation between Cav-1 expression and metabolic alterations during oncogenesis, MCF-10A cells were transfected with the oncogene *RAS* to induce malignant transformation. *RAS-*transformed MCF-10A cells developed more colonies than those of MCF-10A cells, but this was partially reversed by the glycolytic inhibitor 3-BrPA (Supplementary Fig. 1A, B). More importantly, Cav-1 expression and mitochondrial membrane potential were significantly decreased after *RAS* transfection, and this could be partially reversed by 3-BrPA (Supplementary Fig. 1C, D). Similarly, Cav-1-specific siRNAs decreased the mitochondrial membrane potential, impaired mitochondrial respiratory function, and activated aerobic glycolysis activity in MCF-10A cells (Fig. 1e and Supplementary Fig. 1D). Furthermore, Cav-1 overexpression enhanced the mitochondrial membrane potential in MCF-7 cells while Cav-1 silencing decreased that in MDA-MB-231 cells (Supplementary Fig. 1E). Moreover, the time course of the target gene responses upon 3-BrPA treatment was investigated. 3-BrPA treatment firstly induced Cav-1 expression in both MCF-7 and MDA-MB-231 cells, followed by a significant attenuation of c-Myc, and the metabolism-related proteins including LDH-A, PGC-1α and Nrf-1 changed lastly (Fig. 1f). Altogether, these results indicate that Cav-1 may modulate c-Myc and its downstream metabolism-related proteins, and therefore plays a critical role in modulating aerobic-glycolysis activity during breast carcinogenesis.

### Cav-1 limits the self-renewal capacity and aerobic glycolysis activity of BCSCs in vitro

**Fig. 2:**
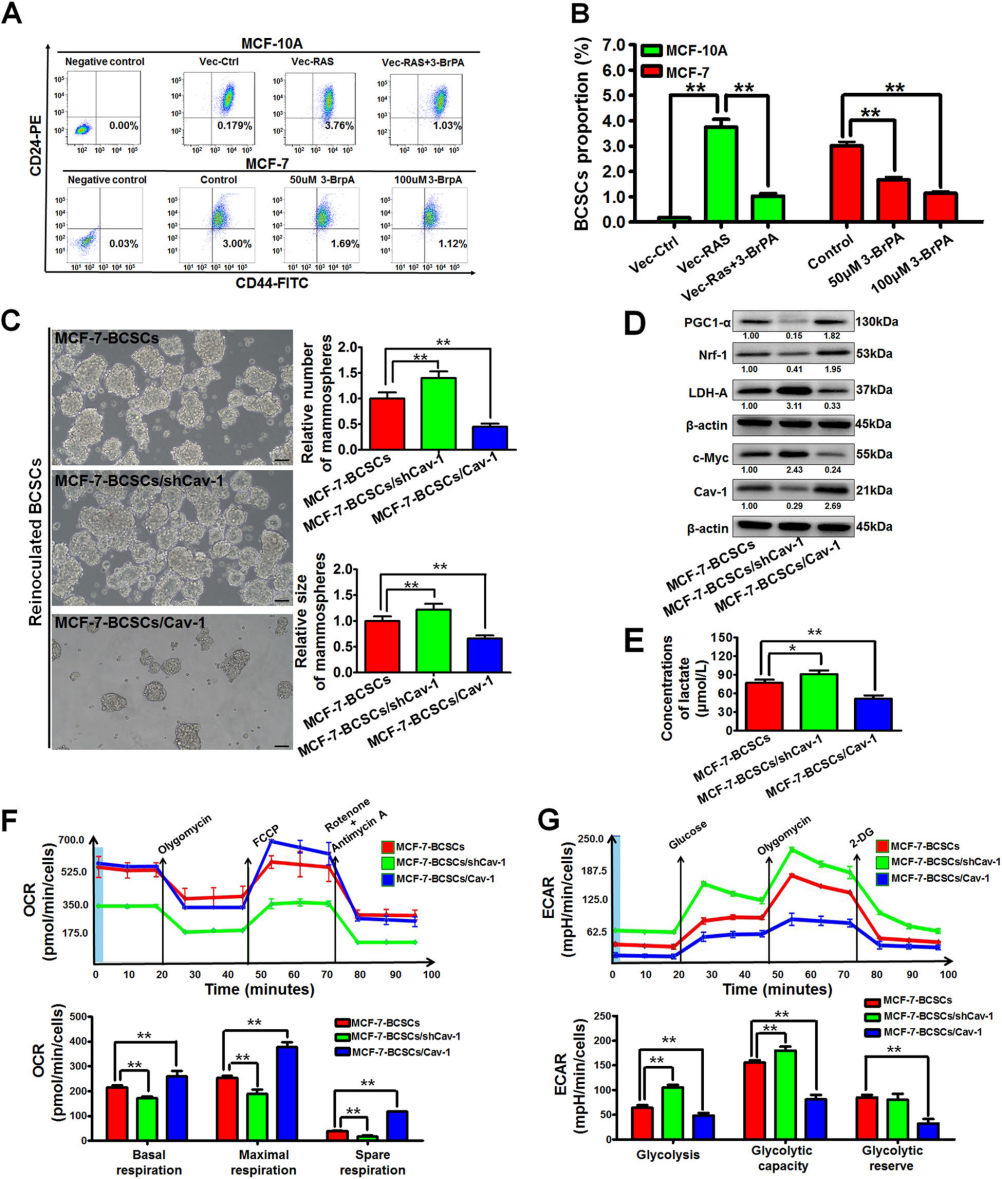
Cav-1 limits the self-renewal capacity and inhibits aerobic glycolysis activity of BCSCs in vitro. **a**, **b** The proportion of BCSCs in MCF-10A cells was significantly increased after *RAS* transfection while 3-BrPA (50 μM) partially reversed this increase. 3-BrPA significantly decreased the proportion of BCSCs in MCF-7 cells. The histogram represents the quantitative analysis of proportions of BCSCs in different groups. *n* = 3. **c** Cav-1 silencing in the reinoculated BCSCs significantly increased not only the number but also the size of mammospheres, whereas Cav-1 overexpression limited the number and size of mammospheres. Scale bar = 100 μm. The histogram represents the quantitative analysis of the numbers and sizes of mammospheres. *n* = 3. **d** Cav-1 knockdown in the reinoculated BCSCs attenuated the expression of mitochondrial respiration-related proteins and elevated the expression of glycolysis-associated proteins, whereas Cav-1 overexpression achieved opposite effects. *n* = 3. **e** Cav-1 knockdown increased lactate production in the reinoculated BCSCs while Cav-1 overexpression decreased that. *n* = 3. **f**, **g** Cav-1 stimulated mitochondrial respiration under conditions of basal mitochondrial respiration, maximal mitochondrial respiration, and spare mitochondrial respiration in reinoculated BCSCs. Meanwhile, Cav-1 could diminish aerobic glycolysis under conditions of glycolysis and glycolytic capacity in reinoculated BCSCs. *n* = 3. All values are presented as the mean ± SD, *P < 0.05, **P < 0.01.

BCSCs are considered as the root of mammary tumorigenesis and development^21^. Therefore, we further investigated the influence of Cav-1 on CD44^+^/CD24^−/low^ BCSCs^22,23^. The proportion of BCSCs in MCF-10A cells was significantly increased after *RAS* transformation, and this could be partially reversed by 3-BrPA (*P* < 0.01). Compared with MCF-10A cells, MCF-7 cells had increased populations of BCSCs, which were also significantly decreased by 3-BrPA (*P* < 0.01) (Fig. 2a, b). These results indicate that inhibition of aerobic glycolysis is a feasible strategy for eliminating BCSCs. BCSCs are enriched in non-adherent spherical clusters of cells, which are termed mammospheres^20^. As shown in Fig. 2c, Cav-1 knockdown in the reinoculated BCSCs significantly increased not only the number but also the size of mammospheres, whereas Cav-1 overexpression limited that. Furthermore, Cav-1 knockdown significantly increased the CD44^+^/CD24^−/low^ subpopulation in the breast cancer xenograft formed by the reinoculated BCSCs, whereas Cav-1 overexpression decreased this subpopulation (Supplementary Fig. 2A, B). These results indicated that Cav-1 could inhibit the self-renewal capacity of reinoculated BCSCs. Moreover, Cav-1 knockdown in the reinoculated BCSCs could attenuate the expression of mitochondrial respiration-related proteins and elevate the expression of glycolysis-associated proteins, whereas Cav-1 overexpression achieved opposite effects (Fig. 2d). Increased lactate production is an important feature of glycolysis activation. Cav-1 knockdown increased lactate production in the reinoculated BCSCs, while Cav-1 overexpression decreased that (Fig. 2e). More convincingly, Cav-1 knockdown impaired the mitochondrial respiration under conditions of basal mitochondrial respiration, maximal mitochondrial respiration, and spare mitochondrial respiration in reinoculated BCSCs, whereas Cav-1 overexpression achieved opposite effects (Fig. 2f). Mitotracker-red staining results also recapitulated these results (Supplementary Fig. 2C, D). In contrast, Cav-1 knockdown stimulated aerobic glycolysis under conditions of glycolysis and glycolytic capacity whereas Cav-1 overexpression achieved opposite effects (Fig. 2g). Altogether, Cav-1 inhibits the self-renewal capacity and aerobic glycolysis activity of BCSCs.

### Cav-1 null mice exhibit accelerated ductal genesis and increased proportions of stem cells in mammary glands in vivo

**Fig. 3:**
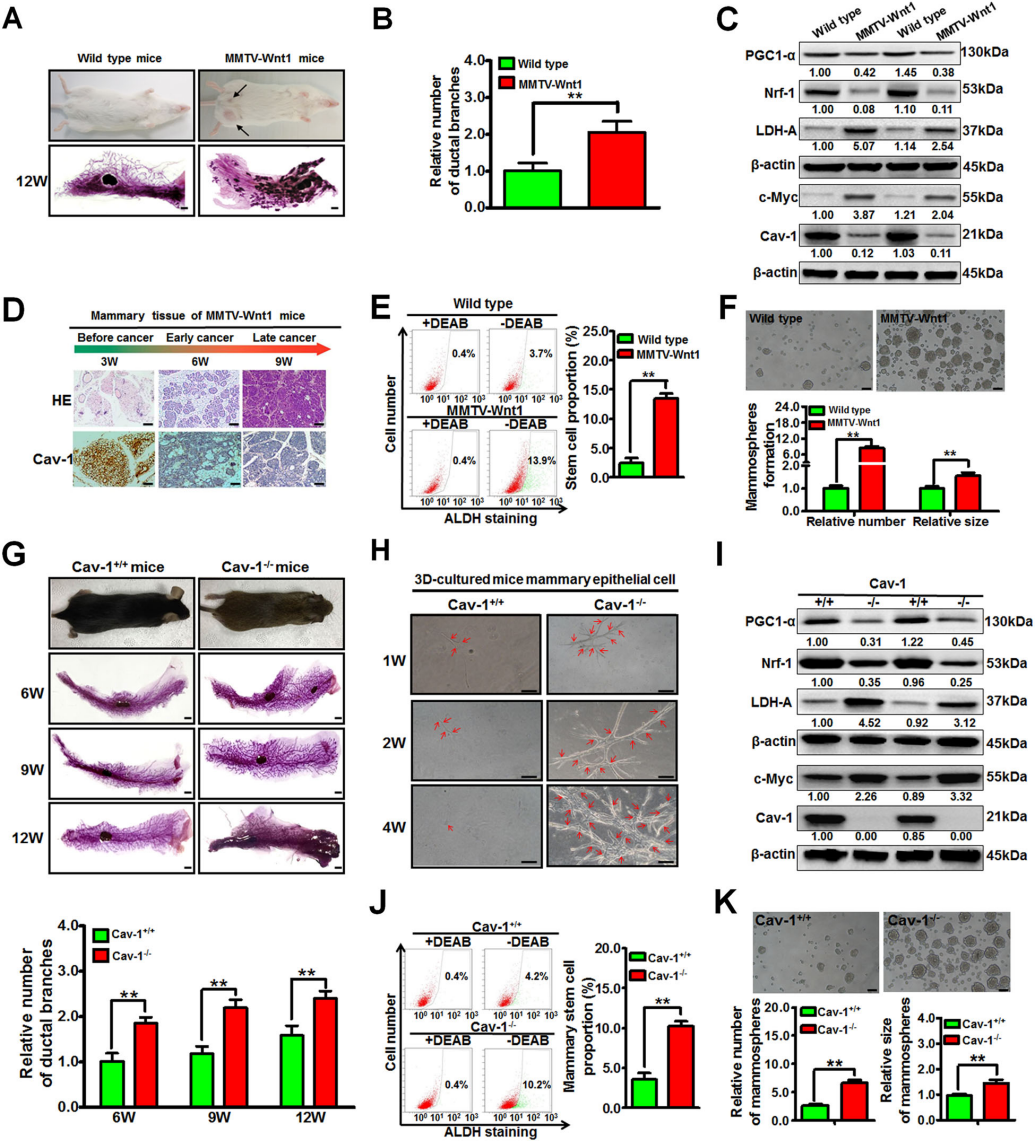
Cav-1 null mice exhibit accelerated ductal hyperplasia and increased proportions of NBSCs in vivo. **a**, **b** Transgenic MMTV-Wnt1 tumor-prone mice formed more ductal oncogenic leisions in mammary glands when compared with those in wild type mice. The histogram represents the quantitative analysis of the numbers of ductal branches. Scale bar = 1 mm, *n* = 12. **c** The mammary glands isolated from MMTV-Wnt1 mice exhibited attenuated expression of Cav-1, PGC1-α, and Nrf-1 when compared with those of wild type mice, accompanied by increased expression of c-Myc and LDH-A. *n* = 3. **d** HE and immunohistochemistry staining of MMTV-Wnt1 mouse mammary-tumor tissues revealed that Cav-1 expression decreased along with the progression of breast cancer. Scale bar = 100 μm. **e** The proportion of ALDH^+^ NBSCs in mammary epithelial cells of MMTV-Wnt1 mice was significantly higher than that of wild type mice. The proportion of NBSCs in mammary gland-derived mammary epithelial cells was analyzed by ALDH staining and flow cytometry. DEAB denotes diethylaminobenzaldehyde. DEAB is used to control for background fluorescence in the ALDH staining assay. *n* = 3. **f** NBSCs isolated from MMTV-Wnt1 mice had increased efficacy to form mammospheres compared to those from wild type mice. Scale bar = 100 μm, *n* = 3. **g** Cav-1^−/−^ mice exhibited an accelerated ductal hyperplasia tendency in mammary glands when compared with that of Cav-1^+/+^ mice. Scale bar = 1 mm, *n* = 12. **h** 3D-cultured mouse mammary epithelial cells isolated from mammary glands of Cav-1^−/−^ mice developed into much more branched structures compared to that of Cav-1^+/+^ mice. Scale bar = 200 μm. **i** Mammary glands isolated from Cav-1^−/−^mice exhibited attenuated expression of Cav-1, PGC1-α, and Nrf-1 when compared with those of Cav-1^+/+^ mice, accompanied by increased expression of c-Myc and LDH-A. *n* = 3. **j** Mammary epithelial cells of Cav-1^−/−^ mice also had a remarkably increased proportion of NBSCs compared with that of Cav-1^+/+^ mice. *n* = 3. **k** Cav-1 loss in NBSCs significantly promoted not only the number but also the size of the mammospheres. Scale bar = 100 μm, *n* = 3. All values are presented as the mean ± SD, **P < 0.01.

Breast cancer usually originates from the uncontrolled proliferation of mammary epithelial cells and mammary-ductal hyperplasia. As shown in Fig. 3a, b, MMTV-Wnt1 mammary-tumor-prone mice developed multiple oncogenic lesions in mammary glands when compared with those of wild type mice (*P* < 0.05), indicating a tendency toward accelerated mammary-ductal hyperplasia. Furthermore, mammary glands isolated from MMTV-Wnt1 mice exhibited decreased Cav-1 expression and elevated aerobic glycolysis activity when compared with that of wild type mice (Fig. 3c). Moreover, Cav-1 expression in MMTV-Wnt1 mice mammary-tumor tissues gradually decreased concomitantly with breast cancer progression (Fig. 3d). Mammary oncogenesis is closely associated with the activity of stem cells. The proportion of ALDH^+^ normal breast stem cells (NBSCs)^23^ in mammary epithelial cells of MMTV-Wnt1 mice was significantly higher than that of wild type mice (*P* < 0.01) (Fig. 3e). Meanwhile, NBSCs isolated from MMTV-Wnt1 mice exhibited increased efficacy to form mammospheres compared to those from wild type mice (*P* < 0.01) (Fig. 3f). Interestingly, similar to MMTV-Wnt1 mice, Cav-1^−/−^ mice also exhibited an accelerated ductal-hyperplasia tendency in mammary glands when compared with that of Cav-1^+/+^ mice (Fig. 3g). This finding is consistent with the findings of Williams et al. in that mammary glands of Cav-1^−/−^ mice exhibited dysregulated ductal hyperplasia^24^. More importantly, 3D-cultured mouse mammary epithelial cells isolated from mammary glands of Cav-1^−/−^ mice developed into much more branched structures compared to that of Cav-1^+/+^ mice (Fig. 3h). These data demonstrated that Cav-1 loss was closely implicated in the pathogenesis of mammary-ductal-hyperplasia. Additionally, mammary glands isolated from Cav-1^−/−^ mice exhibited activated aerobic glycolysis activity and impaired mitochondrial respiration (Fig. 3i). Meanwhile, mammary epithelial cells of Cav-1^−/−^ mice also exhibited a remarkably increased proportion of NBSCs compared with that of Cav-1^+/+^ mice (*P* < 0.01) (Fig. 3j). Cav-1 loss in mouse NBSCs significantly promoted not only the number but also the size of mammospheres (*P* < 0.01) (Fig. 3k). Altogether, Cav-1 deletion is associated with accelerated ductal genesis and increased proportions of NBSCs in mouse mammary glands in vivo.

### Cav-1 loss promotes mouse mammary-tumor formation in vivo by activating aerobic glycolysis activity of BCSCs

**Fig. 4:**
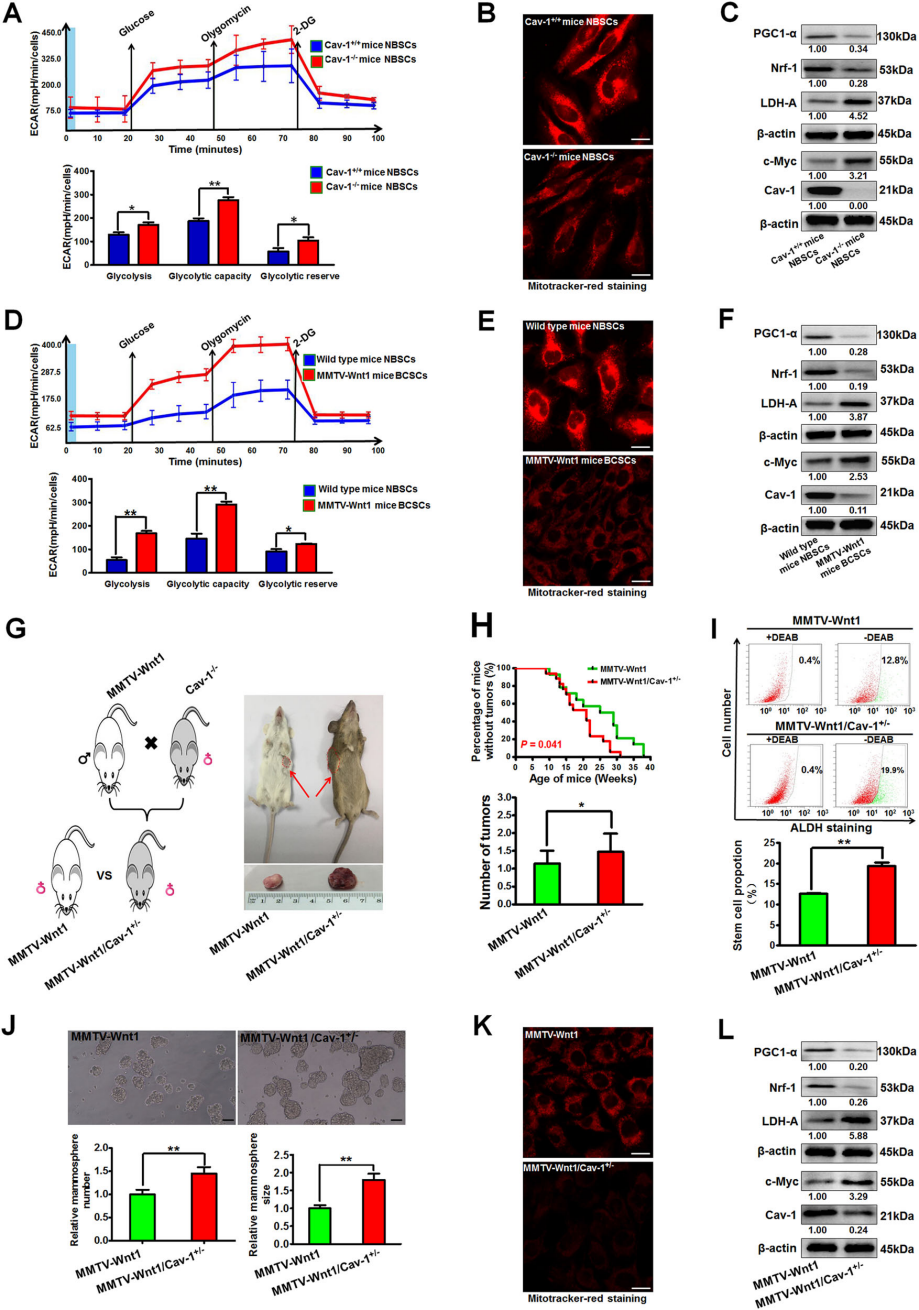
Cav-1 loss promotes mouse mammary-tumor formation in vivo by activating aerobic glycolysis activity of BCSCs. **a–c** NBSCs isolated from Cav-1^−/−^ mice exhibited elevated glycolytic activity (**a**) and impaired mitochondrial membrane potential (**b**) when compared with those isolated from Cav-1^+/+^ mice, which was accompanied by increased expression of glycolysis-related proteins but decreased expression of mitochondrial respiration-associated proteins (**c**). **b** Represents the fluorescent images of mitotracker-red stained cells. Scale bar = 20 μm, *n* = 3. **d–f** BCSCs isolated from mammary tumors of MMTV-Wnt1 mice exhibited elevated glycolytic activity (**d**) and impaired mitochondrial membrane potential (**e**) when compared with NBSCs isolated from wild type mice, which was accompanied by increased expression of glycolysis-related proteins but decreased expression of mitochondrial respiration-associated proteins (**f**). Scale bar = 20 μm, *n* = 3. **g** Transgenic MMTV-Wnt1/Cav-1^+/−^ tumor-prone mice were generated by crossbreeding male MMTV-Wnt1 mice with female Cav-1^−/−^ mice. **h** Female MMTV-Wnt1/Cav-1^+/−^ mice (*n* = 17) developed significantly more breast tumors at an earlier age when compared with those of MMTV-Wnt1 mice (*n* = 14). Kaplan–Meier analyses of tumor-onset time in MMTV-Wnt1 mice and MMTV-Wnt1/Cav-1^+/−^ mice were conducted. **i** The proportion of ALDH^+^ BCSCs in mammary tumors of MMTV-Wnt1/Cav-1^+/−^ mice was significantly higher than that in MMTV-Wnt1 mice. *n* = 3. **j** BCSCs isolated from tumors of the MMTV-Wnt1/Cav-1^+/−^ mice formed more and larger mammospheres than those isolated from tumors of the MMTV-Wnt1 mice. The histogram represents the quantitative analysis of the numbers and sizes of mammospheres. Scale bar = 100 μm, *n* = 3. **k**, **l** Compared with BCSCs isolated from tumors of the MMTV-Wnt1 mice, the BCSCs isolated from tumors of the MMTV-Wnt1/Cav-1^+/−^ mice exhibited decreased mitochondrial membrane potential (**k**), increased expression of glycolysis-related proteins and attenuated expression of mitochondrial respiration-associated proteins (**l**). Scale bar = 20 μm, *n* = 3. All values are presented as the mean ± SD, *P < 0.05, **P < 0.01.

As CSCs may originate from normal stem cells that have been reprogrammed by genetic and/or epigenetic events^25^, the metabolic differences between NBSCs and BCSCs were further compared. NBSCs isolated from Cav-1^−/−^ mice exhibited elevated glycolytic activity and impaired mitochondrial membrane potential when compared with that isolated from Cav-1^+/+^ mice (Fig. 4a–c). Similarly, BCSCs isolated from mammary tumors of MMTV-Wnt1 mice also exhibited elevated glycolytic activity and decreased mitochondrial membrane potential when compared with NBSCs isolated from wild type mice, which was accompanied by decreased expression of Cav-1 but increased expression of c-Myc (Fig. 4d–f). Further studies indicated that decreased Cav-1 expression was not specific to BCSCs but acted as a potential contributor to facilitating stemness (Supplementary Fig. 3).

Given that Cav-1^−/−^ mice could not generate mammary tumors spontaneously, to further investigate the influence of Cav-1 on mice mammary-tumor formation in vivo, MMTV-Wnt1/Cav-1^+/−^ tumor-prone mice were generated by crossbreeding male MMTV-Wnt1 mice with female Cav-1^−/−^ mice (Fig. 4g). The female MMTV-Wnt1/Cav-1^+/−^ mice developed more breast tumors (1.47 tumors vs. 1.14 tumors, *P* < 0.05) at an earlier age (19.6 weeks vs. 24.5 weeks, *P* < 0.05) compared with that of parental MMTV-Wnt1 mice (Fig. 4h). Similarly, two independent studies reported that Cav-1 knockout in mammary-tumor-prone mice was associated with an accelerated onset of mammary tumors, with increased multiplicity and tumor burden in the transgenic mammary-tumor-prone mice, but not in wild type mice^10,26^. Our present study indicated that Cav-1 knockdown in mammary-tumor-prone mice was also accompanied by accelerated onset and growth of mammary tumors. Meanwhile, the proportion of ALDH^+^ BCSCs in mammary tumors of MMTV-Wnt1/Cav-1^+/−^ mice was significantly higher than that of MMTV-Wnt1 mice (*P* < 0.01) (Fig. 4i). Cav-1 excalation in mouse BCSCs also significantly promoted not only the number but also the size of mammospheres (*P* < 0.01, Fig. 4j). Additionally, Cav-1 excalation in mouse BCSCs led to impaired mitochondrial membrane potential and increased glycolytic activity (Fig. 4k, l). These results demonstrate that Cav-1 loss may be the concomitant event accelerating mammary cause for tumorigenesis. Altogether, Cav-1 loss could accelerate mouse mammary-tumor formation in vivo by activating the aerobic glycolysis activity of BCSCs.

### Cav-1 promotes VHL-mediated ubiquitination and degradation of c-Myc in BCSCs through the proteasome pathway

**Fig. 5:**
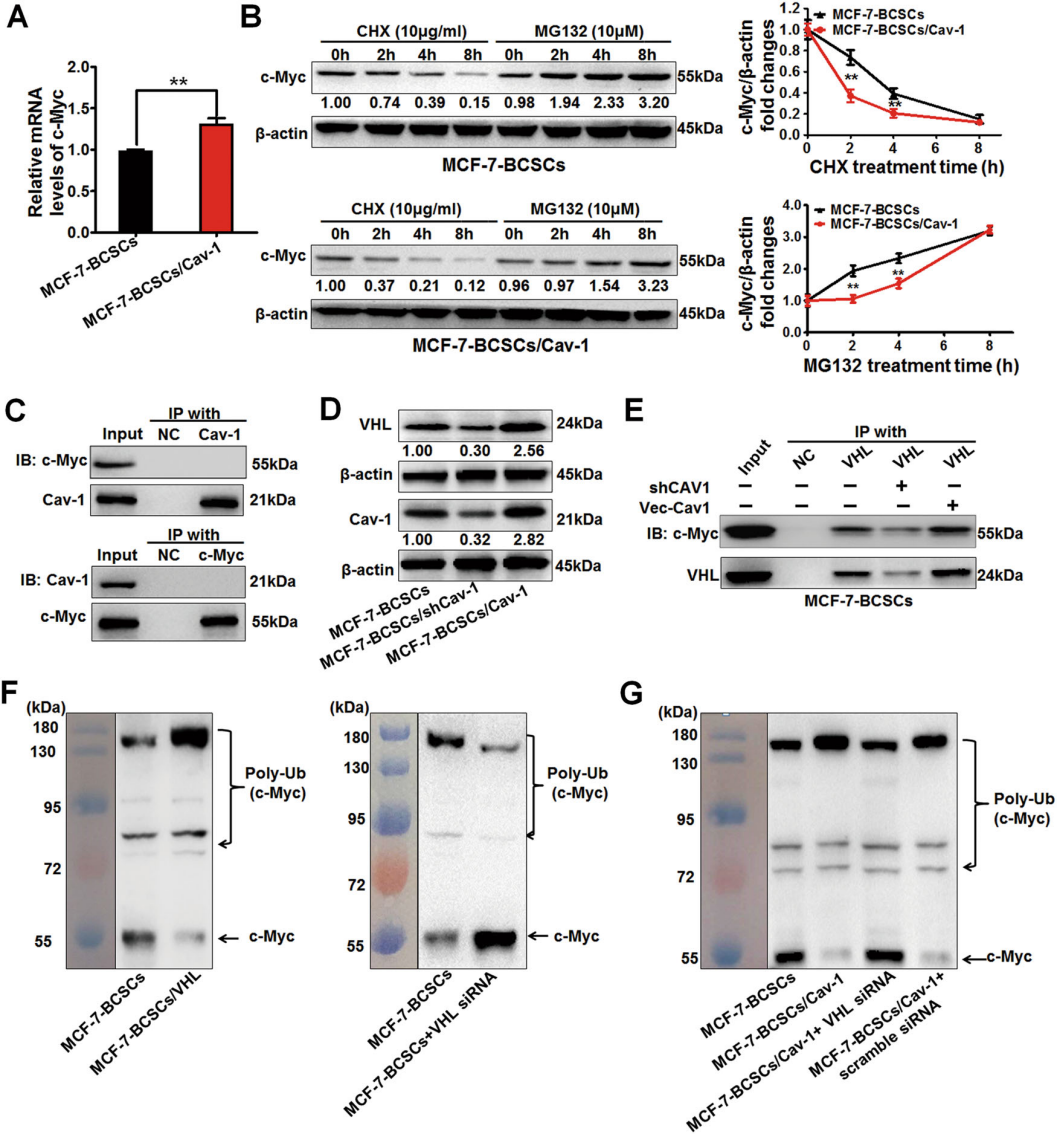
Cav-1 promotes VHL-mediated ubiquitination and degradation of c-Myc in BCSCs through the proteasome pathway. **a** QPCR results showed that the *MYC* mRNA level in BCSCs was significantly elevated after Cav-1 overexpression. *n* = 3. **b** Cav-1 overexpression in BCSCs led to accelerated degradation of c-Myc when the protein-synthesis inhibitor, cycloheximide (CHX, 10 μg/ml), was administrated. Meanwhile, the proteasome inhibitor, MG132 (10 μM), reversed Cav-1 overexpression-accelerated c-Myc degradation in BCSCs. *n* = 3. **c** Immunoprecipitation results indicated that there was no interaction between Cav-1 and c-Myc. NC: negative control, coupling buffer immobilization group without antibody added. *n* = 3. **d** Western blotting results showed that Cav-1 overexpression in BCSCs could induce the up-regulation of VHL, while Cav-1 knockdown could attenuate VHL expression. *n* = 3. **e** Cav-1 overexpression enhanced the interaction between VHL and c-Myc in BCSCs while Cav-1 knockdown weakened this interaction. *n* = 3. **f** VHL overexpression could induce the ubiquitination of c-Myc in BCSCs while VHL knockdown could inhibit that. *n* = 3. **g** Cav-1 induced the ubiquitination of c-Myc in BCSCs while VHL-specific siRNAs restored this process. *n* = 3. All values are presented as the mean ± SD, **P < 0.01.

Based on the clarification regarding the modulatory effect of Cav-1 on metabolic reprogramming in BCSCs, it is necessary to elucidate its underlying molecular mechanisms. Metabolic alterations in tumors are coordinated by multiple genes, the most prominent of which is the oncogene *MYC* due to its extensive transcriptional modulatory effects^27^ on glycolysis rate-limiting enzymes including hexokinase 2 (HK2) and PKM2^28^. As indicated above, Cav-1 attenuated c-Myc expression in multiple in vitro and in vivo assays. However, Cav-1 overexpression elevated *MYC* mRNA levels in BCSCs (Fig. 5a), indicating that Cav-1 might attenuate c-Myc expression at the posttranscriptional level. The ubiquitin–proteasome system (UPS) is the most prominent pathway for modulation of cellular c-Myc protein homeostasis^29^. Cav-1 overexpression in BCSCs led to accelerated degradation of c-Myc while MG132, a proteasome inhibitor, could reverse that (Fig. 5b). These results suggested that Cav-1 could accelerate the degradation of c-Myc in BCSCs through the proteasome pathway. There was no interaction between Cav-1 and c-Myc, suggesting that Cav-1 may indirectly modulate the degradation process of c-Myc (Fig. 5c). Accumulating studies have reported that VHL, a well-known E3 ubiquitin ligase and tumor suppressor protein, could mediate the ubiquitination and degradation of hypoxia-inducible factor (HIF)^30^. Our studies also suggested that Cav-1 could accelerate the degradation of HIF1α which might be mediated by upregulating VHL (Supplementary Fig. 4A, B, Fig. 5d). Therefore, we further investigated whether Cav-1 also induced the degradation of c-Myc through VHL-mediated ubiquitin–proteasome system. Co-IP results demonstrated that Cav-1 overexpression in BCSCs enhanced the interaction between VHL and c-Myc while Cav-1 knockdown weakened this interaction (Fig. 5e). Meanwhile, VHL overexpression induced the ubiquitination of c-Myc in BCSCs whereas VHL silencing inhibited this process (Fig. 5f). More importantly, Cav-1 overexpression in BCSCs induced the ubiquitination of c-Myc, while VHL-specific siRNAs restored this process (Fig. 5g). Altogether, Cav-1 could promote VHL-mediated ubiquitination and degradation of c-Myc in BCSCs through the proteasome pathway.

### Cav-1 predicts a better clinical outcome in breast cancer patients

**Fig. 6:**
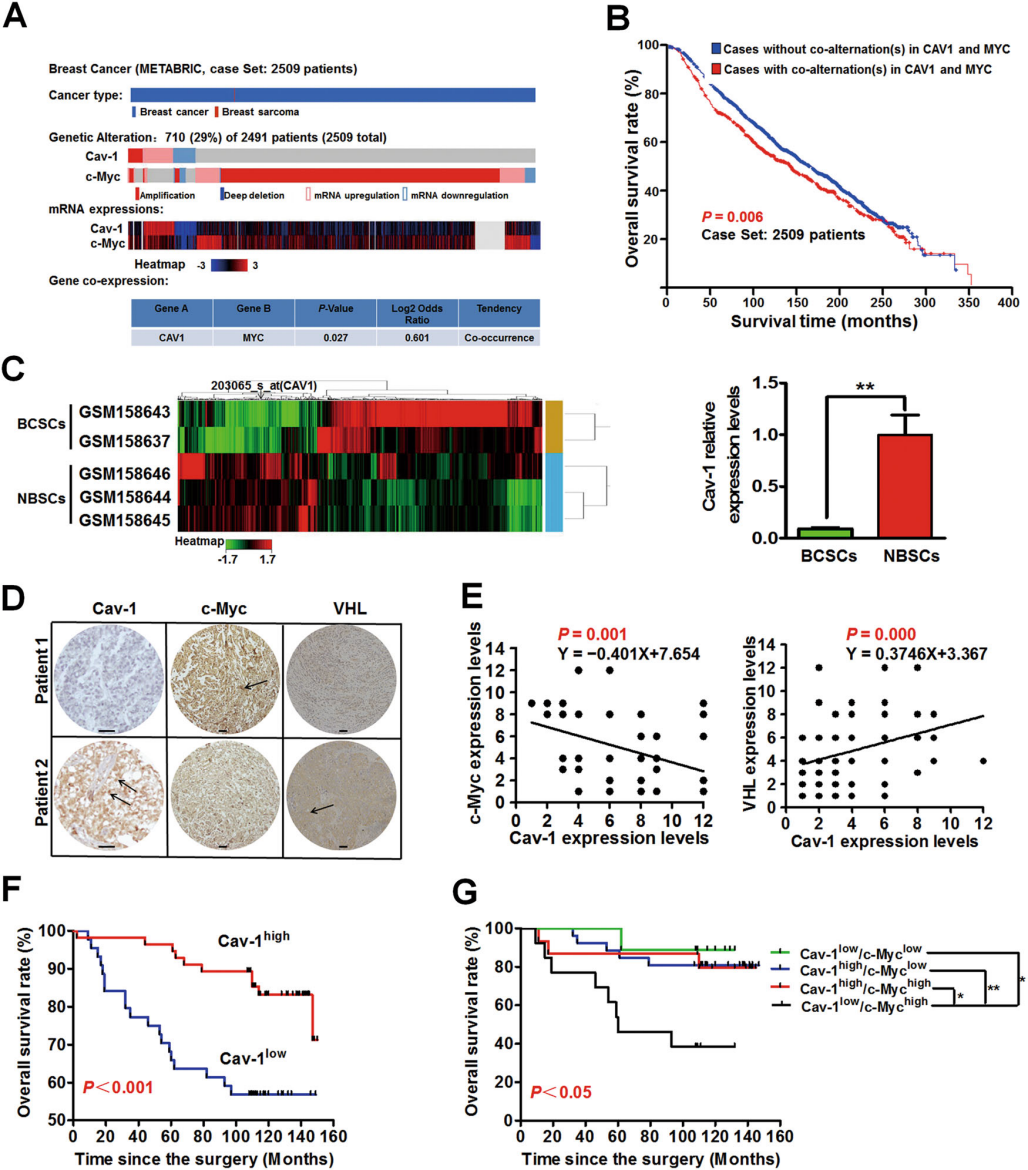
Cav-1 predicts a better clinical outcome in breast cancer patients. **a** There was a significant tendency toward co-occurrence between alterations in *CAV1* and alterations in *MYC*. Co*-*alternation(s) of *CAV1* and *MYC* in 710 (29%) of 2491 sequenced cases/patients. Case Set: METABRIC, Nature 2012 & Nat Commun 2016. Log odds ratio > 0: Association toward co-occurrence. *P* < 0.05: Significant association. *n* = 2509. **b** Cases with co-alterations in *CAV1* and *MYC* predicated a poorer overall survival time compared with that of the cases without co-alterations in *CAV1* and *MYC*. Deaths from all causes were analyzed. *n* = 2509. **c** The relative expression level of *CAV1* gene in tumorigenic BCSCs was significantly decreased when compared with that of NBSCs. *CAV1* gene expression profiles of BCSCs and NBSCs were compared (left) and analyzed (right). *n* = 24. **d** Representative pictures of Cav-1 expression, c-Myc expression and VHL expression in cancer tissues of human breast cancer patients. Scale bar = 50 μm. **e** Correlation analysis validated the negative correlation between Cav-1 and c-Myc expression levels (*n* = 63), as well as the positive correlation between Cav-1 and VHL expression levels (*n* = 121) in breast cancer patients. Correlation analysis between expression levels of indicated proteins was conducted using human breast cancer tissue microarray and Spearman rank correlation analysis. **f** Human breast cancer tissue microarray analysis indicated that Cav-1 expression was positively associated with a better overall survival in breast cancer patients (*P* < 0.001). *n* = 100. **g** Joint analysis suggested that patients with Cav-1^low^/c-Myc^high^ phenotype exhibited the worst overall survival. *n* = 63. *P < 0.05, **P < 0.01.

Lastly, the clinical implications of Cav-1 in breast cancer were analyzed. Bioinformatics analysis^31^ indicated that there was a significant tendency toward co-occurrence between alterations in *CAV1* and alterations in *MYC* in breast cancer patients (*P* = 0.027, Fig. 6a). Meanwhile, cases with co-alterations in *CAV1* and *MYC* predicated a poorer overall survival compared with that of cases without co-alterations in *CAV1* and *MYC* (*P* = 0.006, Fig. 6b). Furthermore, Cav-1 expression was significantly decreased in tumorigenic BCSCs when compared with that of NBSCs (*P* < 0.01, Fig. 6c). Moreover, the correlation between epithelial Cav-1 expression and clinicopathological parameters of breast cancer patients was analyzed using a human breast cancer tissue microarray (Fig. 6d). Correlation analysis validated the negative correlation between Cav-1 and c-Myc expression levels (*P* = 0.001) as well as the negative correlation between Cav-1 and HIF1α expression levels (*P* = 0.018) in breast cancer patients. In contrast, a positive correlation between Cav-1 and VHL expression levels (*P* = 0.000) were observed (Fig. 6e and Supplementary Fig. 4C, D). Importantly, high epithelial Cav-1 expression was associated with a better overall survival (*P* < 0.001) and a delayed cancer onset time (*P* < 0.05) (Fig. 6f and Table 1). Meanwhile, the joint analysis results suggested that patients with Cav-1^low^/c-Myc^high^ or Cav-1^low^/HIF1α^high^ phenotype exhibited the worst overall survival (Fig. 6g and Supplementary Fig. 4E). Additionally, multivariate Cox regression analysis (Supplementary Table 1) showed that a low expression level of epithelial Cav-1 was an independent hazard for overall survival in breast cancer patients (*P* = 0.002), strongly suggesting epithelial Cav-1 as a potential prognostic marker for breast cancer. Altogether, Cav-1 predicts a better clinical outcome in breast cancer patients.

**Table 1:** Correlation between Cav-1 expression and clinicopathologic parameters of breast cancer.

Parameters	Cases (N)	Cav-1 expression levels		*P* value
		Low (%)	High (%)	
Survival time (months)				
<106	25	19 (76.0%)	6 (24.0%)	<0.001**
≥106	75	25 (33.3%)	50 (66.7%)	
Age (years)				
<54	57	29 (50.9%)	28 (49.1%)	0.017**
≥54	43	15 (34.9%)	28 (65.1%)	
Pathological types				
Luminal A				
Yes	35	13 (37.1%)	22 (62.9%)	
No	52	23 (44.2%)	29 (55.8%)	0.516
Unclear	13	8 (61.5%)	5 (38.5%)	
Luminal B				
Yes	21	8 (38.1%)	13 (61.9%)	
No	66	28 (42.4%)	38 (57.6%)	0.729
Unclear	13	8 (61.5%)	5 (38.5%)	
HER2-overexpressed				
Yes	13	5 (38.5%)	8 (61.5%)	
No	74	31 (41.9%)	43 (58.1%)	0.819
Unclear	13	8 (61.5%)	5 (38.5%)	
Triple-negative				
Yes	18	10 (55.6%)	8 (44.4%)	
No	69	26 (37.7%)	43 (62.3%)	0.174
Unclear	13	8 (61.5%)	5 (38.5%)	
Clinical stage				
Tumor status (T)				
T1	24	9 (37.5%)	15 (62.5%)	
T2	65	31 (47.7%)	34 (52.3%)	0.759
T3	11	4 (36.4%)	7 (63.6%)	
Lymph node status (N)				
N0	38	16 (42.1%)	22 (57.9%)	
N1	30	15 (50.0%)	15 (50.0%)	
N2	24	10 (41.7%)	14 (58.3%)	0.834
N3	6	3 (50.0%)	3 (50.0%)	
Unclear	2	0 (0.00%)	2 (100%)	

**p < 0.05, *n* = 100.

## Discussion

Extensive efforts are being made to identify and develop novel targets or treatment strategies for breast cancer. BCSCs are considered to be the root of breast cancer occurrence and progression. While most existing CSCs studies have focused on stemness-modulatory signaling, few studies have focused on the subcellular structure and membrane composition of CSCs^7,8^. Since biomolecules in membrane rafts play important roles in mediating responses to external stimuli, signal acquisition and crosstalk, and the activation of downstream pathways^32^, it is of great interest to explore the differences in membrane compositions between malignant CSCs and normal stem cells. Cav-1 is a constituent protein of plasma membrane. Herein, we demonstrated that downregulation of Cav-1 occurred in BCSCs when compared with that of NBSCs, and was associated with a metabolic switch from mitochondrial respiration to aerobic glycolysis in BCSCs. Additionally, Cav-1 inhibits the self-renewal capacity and aerobic glycolysis activity of BCSCs. Therefore, a Cav-1-resurrection strategy in BCSCs may be a potential treatment strategy for breast cancer. Since Cav-1 is an integral membrane protein of caveolae, we speculate that a nano-drug delivery system loaded with recombinant Cav-1 protein may be a potential strategy to transfer Cav-1 into caveolae of BCSCs by cytomembrane endocytosis and therefore inhibit the self-renewal capacity of BCSCs. In addition, a mammary gland-specific expressional vector of the *CAV1* gene may selectively elevate Cav-1 expression both in the stroma and in BCSCs and, therefore, may act as a potential gene treatment strategy for both malignant transformation prevention and BCSCs elimination in the future. Besides, it may be relatively easy to develop Cav-1-targeted drugs to inhibit breast cancer growth. In the present study, we found that 3-BrPA could induce Cav-1 expression and therefore inhibit the BCSCs self-renewal in *RAS*-transformed MCF-10A cells and breast cancer MCF-7 cells. It has also been reported that 3-BrPA treatment alone showed excellent antitumor activities in breast cancer xenografts and could sensitize breast cancer tumor to chemotherapy via reprogramming cancer metabolism^33,34^. What is more, our previous study identified betulinic acid as an inducer of Cav-1 expression in breast cancer cells^35^. It could significantly inhibit breast cancer growth and glycolytic activity in both the transgenic MMTV-PyVT^+/−^ breast cancer spontaneous model and the zebrafish breast cancer xenotransplantation model in vivo by upregulating Cav-1 expression^35^. Herein, betulinic acid treatment significantly elevated Cav-1 expression whereas attenuated the stemness-related protein ALDH1A1 in mammary tumors of MMTV-PyVT^+/-^ breast cancer spontaneous mice (Supplementary Fig. 5). These results indicated Cav-1 may be a druggable target for BCSCs elimination and breast cancer treatment. Considering that Cav-1 downregulation was associated with a poor overall survival and an accelerated breast cancer onset, we speculate that Cav-1 may become a diagnostic and prognostic biomarker for breast cancer patients in the future.

Interestingly, Cav-1 overexpression was also implicated in the pathogenesis of oncogenic transformation and tumorigenesis in bladder and prostate carcinomas^36^. The different roles of Cav-1 in modulating oncogenic transformation in different kinds of tumors have been attributed to its fluctuating expression in different organs and stages^37^. Because of the heterogeneity of Cav-1 expression in different tumors, therapies targeting Cav-1 will need to consider the specific roles or functions of this protein within the corresponding type of tumor cell. Additionally, Cav-1 also acts as a stress signal in multiple cancer cells, and various microenvironmental factors could also be efficient in modulating Cav-1 expression levels. For example, hypoxia could induce Cav-1 protein levels in a HIF1α-dependent manner in murine melanoma and colon-adenocarcinoma cells^38^. Hypoxic regions of hepatocellular-carcinoma xenografts displayed elevated expression of Cav-1^39^. In addition, hypoxia was a determinant of CSCs evolution and could promote the generation and maintenance of CSCs^40^. However, the underlying molecular network between microenvironmental factors and Cav-1 induction still remains unclear. As Cav-1 downregulation could bring metabolic adaptations within BCSCs, further research is warranted to investigate the underlying mechanisms of Cav-1 downregulation in breast CSCs under hypoxic conditions, which will contribute to a better understanding of the relationship between Cav-1 and metabolic regulation.

It is interesting to note that several independent studies have suggested a close dependence between the undifferentiated status and aerobic glycolysis phenotype of cells, which has been termed the “metabolic state hypothesis.” For example, Prigione et al. reported that undifferentiated stem cells exhibited reduced mitochondrial function and stronger dependence on aerobic glycolysis compared with that of fully differentiated cells^41^. Chen et al. showed that mitochondrial biogenesis function was recovered during the differentiation process of multiple stem cells (i.e., embryonic and hematopoietic stem cells)^42^. Until now, information on the metabolic characteristics of CSCs has been very limited. Ye et al. reported that lung CSCs have a lower oxygen consumption rate and ROS level when compared with those in non-CSCs^43^. Lagadinou *et al*. demonstrated that leukemia CSCs exhibited lower ROS levels and reduced oxidative phosphorylation when compared with non-CSCs^44^. In the present study, we demonstrated that BCSCs exhibited activated aerobic glycolysis activity and impaired mitochondrial respiration when compared with that in NBSCs. Based on the above evidence, we speculate that aerobic glycolysis might be developed as a tool for CSCs recognition and combating cellular-fate decisions in the future.

Understanding the molecular interplay between Cav-1 and metabolic modulation could help uncover druggable metabolic targets or pathways for cancer therapy. Therefore, we investigated how Cav-1 variation induced robust changes in cellular metabolism. Metabolic alterations in tumors are coordinated by multiple genes, prominent among which is the oncogene *MYC*, which encodes the transcription factor c-Myc^27^. Our results indicated that the influence of Cav-1 on c-Myc regulation might occur at the level of post-translational modification and degradation processing. The most prominent route for cellular c-Myc degradation is through the UPS^29^. VHL, a well-known E3 ubiquitin ligase and tumor suppressor protein, mediates the ubiquitination and degradation of many proteins, including HIF^30^. Herein, our study firstly reported that Cav-1 could promote VHL-mediated ubiquitination and subsequent degradation of c-Myc through the proteasome pathway. This finding is consistent with the results of Gordan et al. that VHL loss in clear cell renal carcinomas displayed elevated c-Myc activity^45^ as well as with the findings of Johanne et al. that mutations in VHL could drive aerobic glycolysis in renal cell cancer^46^. Recent studies have suggested that Cav-1 might elevate VHL expression by inducing its transcription through the FOXO3 pathway^47,48^. However, further studies are still needed to determine if this potential mechanism also applies to BCSCs.

**Fig. 7:**
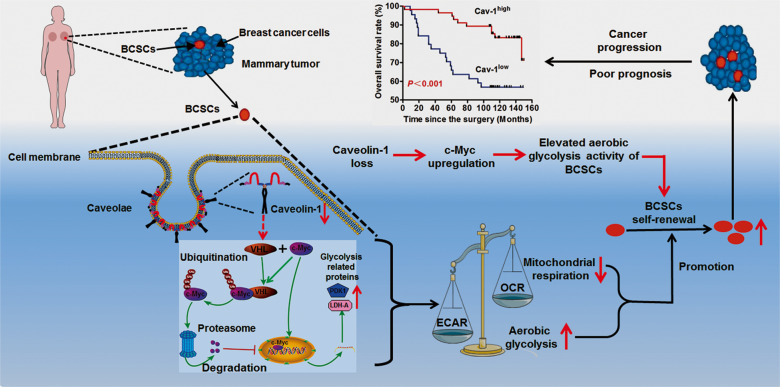
The schematic diagram of the mechanism by which Cav-1 inhibits BCSCs. Downregulation of Cav-1 usually occurred in BCSCs and was associated with a metabolic switch from mitochondrial respiration to aerobic glycolysis. Elevated aerobic glycolysis activity in BCSCs could promote the self-renewal capacity of BCSCs and further lead to poor overall survival of breast cancer patients.

In summary, our findings not only elucidate metabolic characteristics of BCSCs and the inhibitory role of Cav-1 in BCSCs, but also uncover the novel function of Cav-1 in suppressing c-Myc-induced metabolic reprogramming in BCSCs through the ubiquitin–proteasome pathway (Fig. 7). Our study sheds novel light on Cav-1 targeted metabolic modulation as a promising strategy for breast cancer treatment *via* BCSCs elimination.

## Materials and methods

### Cell lines

Human cell lines including MCF-10A, MCF-7, MDA-MB-231, SK-BR-3, T47D, and BT-474 were obtained from the American Type Culture Collection. The identities of all these cell lines have been authenticated by short tandem repeat profiling.

### Animal experiments

Transgenic FVB.Cg-Tg(Wnt1)1Hev/J mice (no.002934, Jackson Laboratory; Strain of Origin: C57BL/6 and SJL, also named MMTV-Wnt1 mammary-tumor-prone mice), transgenic Cav-1^−/−^ mice (Cav1^tm1Mls^/J mice; no.004585, Jackson Laboratory; Strain of Origin: STOCK 129/Sv and C57BL/6J and SJL), and corresponding wild type mice were raised at the Experimental Animal Center of Guangzhou University of Chinese Medicine under specific pathogen-free conditions. All in vivo experiments were performed according to our institution’s guidelines for the use of laboratory animals and were approved by the Institutional Animal Care and Use Committee of Guangdong Provincial Hospital of Chinese Medicine (no.2015008). The MMTV-Wnt1/Cav-1^+/−^ mice were generated by crossbreeding male MMTV-Wnt1 mice with female Cav-1^−/−^ mice. The differences in the numbers and onset times of breast tumors between female MMTV-Wnt1 mice and MMTV-Wnt1/Cav-1^+/−^ mice were compared. No blinding methods were needed in the animal experiments. Breast cancer cells were isolated from mouse mammary tumors by mechanical and enzyme digestive methods.

### Human breast cancer tissue microarray

A commercialized human breast cancer tissue microarray (Outdo Biotech, Shanghai, China) was used to analyze the correlation between Cav-1 expression and the clinicopathological parameters of breast cancer patients. Briefly, Cav-1, c-Myc, VHL or HIF1α expressions in samples were detected by immunohistochemistry. Then, epithelial Cav-1, c-Myc, VHL or HIF1α expression levels were assessed and scored by two experienced pathologists using the immune response score (IRS) method. In the IRS scoring system, the staining intensity was scored semi-quantitatively with the number of 0 (no color reaction), 1 (middle reaction), 2 (moderate reaction) and 3 (intense reaction), while the staining area was scored with the number of 0 (no staining), 1 (staining of 10% cells), 2 (staining of 10%~50% cells), 3 (staining of 51%~80% cells) and 4 (staining of more than 80% cells). The final score was calculated by multiplying the area score by the intensity score. Finally, clinicopathological parameters were divided into the indicated groups according to protein expression levels and were compared.

Detailed experimental method descriptions about Cell culture, Western blotting and Immunoprecipitation, Transmission electron microscopy observation, Oxygen consumption rate (OCRs) and extracellular acidification rate (ECARs), Colony formation assay, Transfection of plasmid and siRNA, Mitotracker-red staining, Stem cell population analysis, Sorting and mammosphere formation assay, Lactate concentration detection assay, Mammary whole mounting, HE and immunohistochemistry, Isolation and three-dimensional culture of mice mammary epithelial cells, Immunofluorescence, QPCR, In vitro ubiquitination assay as well as Bioinformatics analysis are provided in the Supplementary Materials and Methods.

### Statistical analysis

All statistical analyses were performed using SPSS 17.0 software (Abbott Laboratories, Chicago, USA). To ensure adequate power to detect a pre-specified effect, the sample size was chosen using the Power and Sample Size Program (http://biostat.mc.vanderbilt.edu/PowerSampleSize). Student’s *t*-test and one-way ANOVA were performed for comparisons among groups. Levene’s Test of Equality of Variances was used to assess the assumption of homogeneity of variance. Overall survival time was calculated from the date of the first curative operation to the date of the last follow-up or death from all causes. Survival curves were calculated using Kaplan–Meier analysis and were compared using log-rank test. Pearson or Spearman’s rank correlation coefficient was used to analyze the correlation between protein expression levels and clinicopathological characteristics. Univariate and multivariate analyses (Cox proportional hazards regression model) were performed to identify the independent factors relevant to patient survival. Data are represented as the mean ± SD from three independent experiments performed in triplicate at least. All tests were 2-sided and *P* values less than 0.05 were considered statistically significant. The statistical analysis results of immunoblotting images were presented in Supplementary Figs. 6–8.

## Supplementary information

Supplementary Table 1

Supplementary Figure 1

Supplementary Figure 2

Supplementary Figure 3

Supplementary Figure 4

Supplementary Figure 5

Supplementary Figure 6

Supplementary Figure 7

Supplementary Figure 8

Supplementary Figure Legends

Supplementary Materials and Methods
