# Supplementary material for: Caveolin-1 inhibits breast cancer stem cells via c-Myc-mediated metabolic reprogramming
Source: Cell Death Dis. 2020 Jun 11;11(6):450. doi: 10.1038/s41419-020-2667-x (PMC7290025; doi:10.1038/s41419-020-2667-x)
Supplement: Supplementary file 1 — Supplementary Table 1 [file 41419_2020_2667_MOESM1_ESM.doc]

Supplementary Table 1. Prognostic value of Cav-1 for overall survival in breast cancer by univariate and multivariate

analyses

| **Variables** | **Univariate analysis** |  | **Multivariate analysis** | | | | |
| --- | --- | --- | --- | --- | --- | --- | --- |
|  | *p* value | RR | | 95% IC | | | *p* value |
|  |  |  |  | Lower | | Upper |  |
| Tumor Size (T1 + T2 vs. T3) | 0.271 | -- | | -- | | -- | -- |
| HER2 (high vs. low) | 0.214 | -- | | -- | | -- | -- |
| Lymph node infiltration (Yes vs. No) | 0.174 | -- | | -- | | -- | -- |
| Histologic grade (G1 + G2 vs. G3) | 0.170 | -- | | -- | | -- | -- |
| PR (high vs. low) | 0.040^**^ | -- | | -- | | -- | -- |
| Lymph node status (I + II vs. III + IV) | 0.020^**^ | -- | | -- | | -- | -- |
| TNM stage (I + II vs. III ) | 0.018^**^ | -- | | -- | | -- | -- |
| ER (high vs. low) | 0.011^**^ | 0.400 | | 0.171 | | 0.936 | 0.035^**^ |
| Cav-1 (high vs. low) | < 0.001^**^ | 0.258 | | | 0.111 | 0.604 | 0.002^**^ |

^**^*p*< 0.05, statistically significant prognostic factor identified by Univariate/Multivariate analysis. N=100.
