# Supplementary figures and images for: Caveolin-1 inhibits breast cancer stem cells via c-Myc-mediated metabolic reprogramming
Source: Cell Death Dis. 2020 Jun 11;11(6):450. doi: 10.1038/s41419-020-2667-x (PMC7290025; doi:10.1038/s41419-020-2667-x)

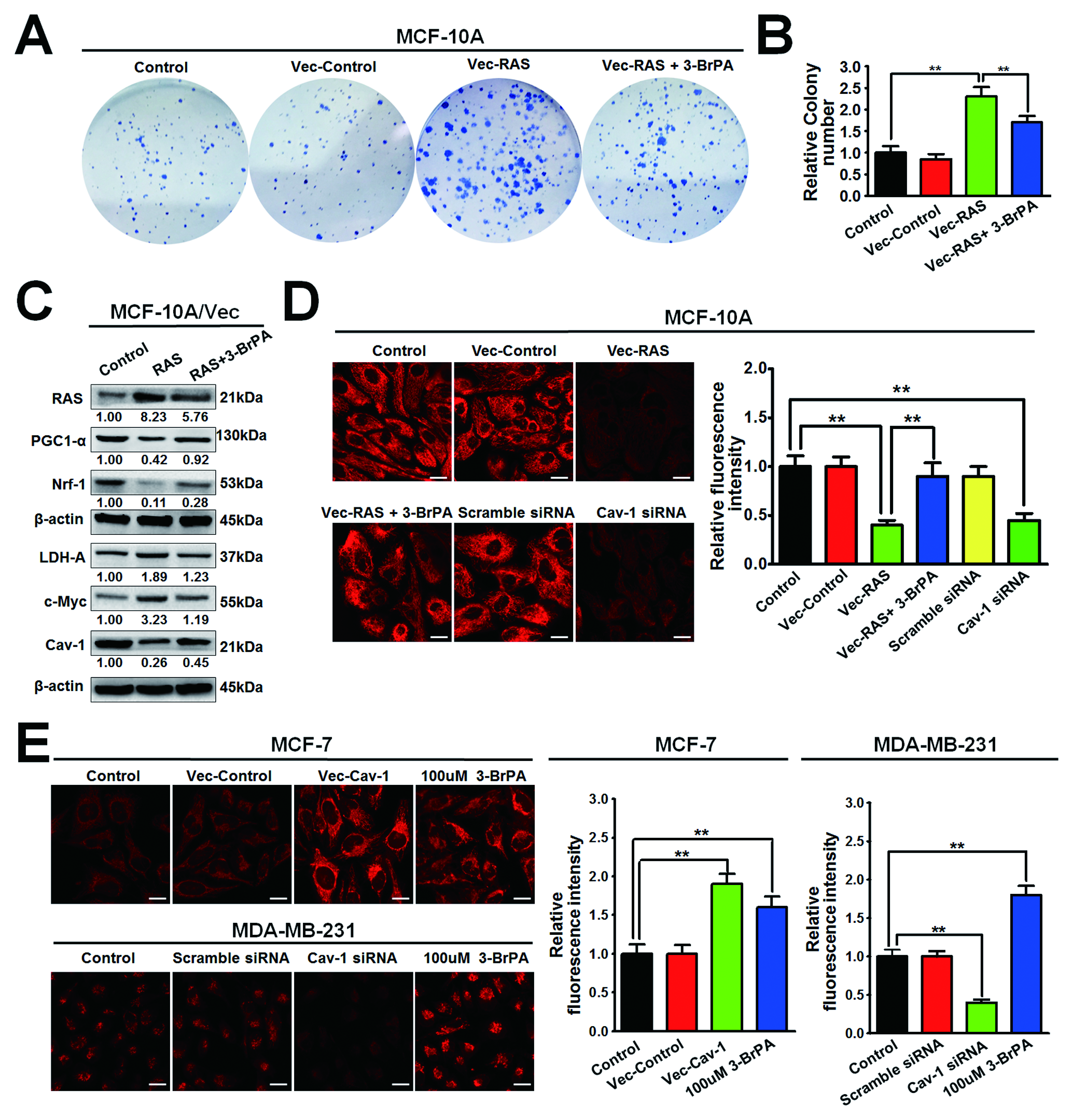

Supplement: Supplementary file 2 — Supplementary Figure 1 [file 41419_2020_2667_MOESM2_ESM.tif]

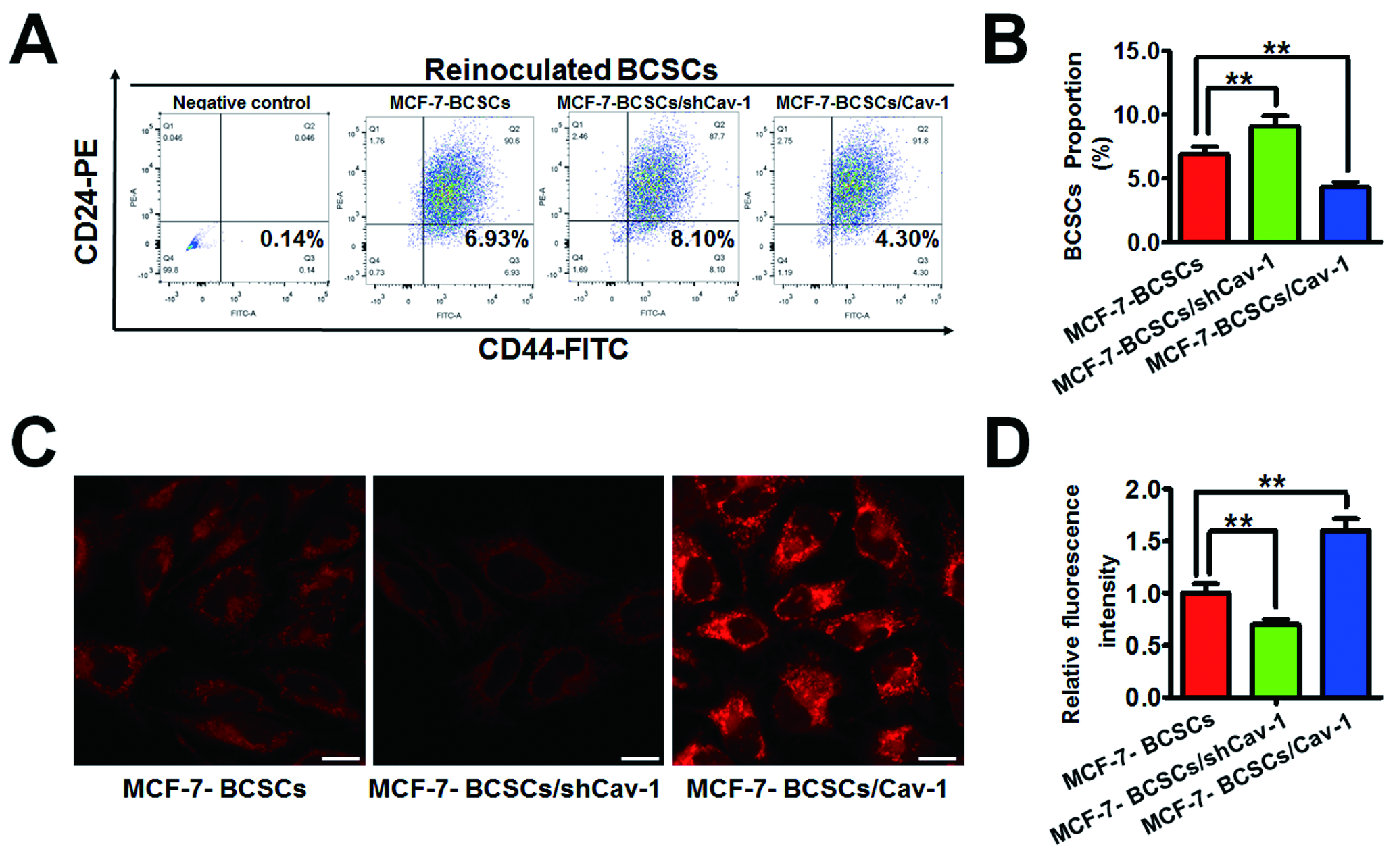

Supplement: Supplementary file 3 — Supplementary Figure 2 [file 41419_2020_2667_MOESM3_ESM.tif]

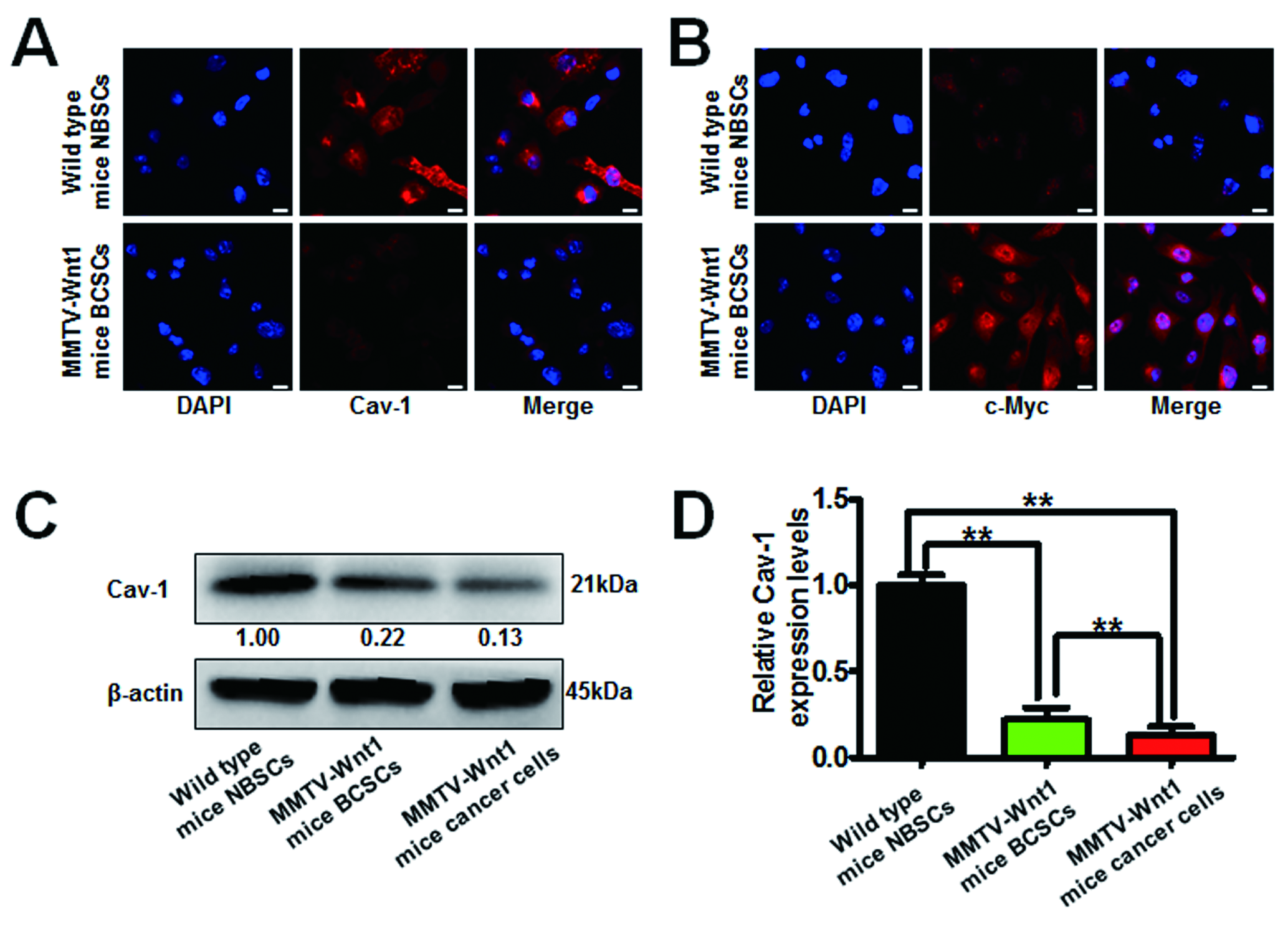

Supplement: Supplementary file 4 — Supplementary Figure 3 [file 41419_2020_2667_MOESM4_ESM.tif]
