# Supplementary material for: Caveolin-1 inhibits breast cancer stem cells via c-Myc-mediated metabolic reprogramming
Source: Cell Death Dis. 2020 Jun 11;11(6):450. doi: 10.1038/s41419-020-2667-x (PMC7290025; doi:10.1038/s41419-020-2667-x)
Supplement: Supplementary file 10 — Supplementary Figure Legends [file 41419_2020_2667_MOESM10_ESM.doc]

**Supplementary Figure Legends**

**Supplementary Figure 1. Cav-1 plays a critical role in modulating aerobic-glycolysis activity during breast carcinogenesis.** (**A-B**) *RAS*-transformed MCF-10A cells developed more colonies than the control group while the glycolysis inhibitor, 3-BrPA (50 μM), could partially reverse that. N = 3. (**C**) *RAS*-transformed MCF-10A cells exhibited decreased expression of Cav-1, Nrf-1 and PGC1-α as well as increased expression of c-Myc and LDH-A, while 3-BrPA (50 μM) could partially reverse that. N = 3. (**D**) Mitotracker-red staining results indicated that the impaired mitochondrial membrane potential of MCF-10A cells after *RAS* transfection could be rescued by 50 μM 3-BrPA. In addition, Cav-1 knockdown impaired mitochondrial membrane potential of MCF-10A cells. The histogram (right) represents the quantitative analysis of the fluorescence intensities of mitotracker-red-stained cells. Scale bar = 20 μm. N = 3. (**E**) Cav-1 overexpression elevated the mitochondrial membrane potential in MCF-7 cells, while Cav-1 knockdown impaired that in MDA-MB-231 cells. Besides, 3-BrPA (100 μM) could elevate mitochondrial membrane potential in both MCF-7 and MDA-MB-231 cells. The histogram represents the quantitative analysis of the fluorescence intensities of mitotracker-red-stained cells. Scale bar = 20 μm. N = 3. All values are presented as the mean ± SD, ^**^*P* < 0.01.

**Supplementary Figure 2. Effects of Cav-1 on the subpopulation and mitochondrial respiration function of BCSCs.** (A-B) Cav-1 knockdown significantly increased the populations of BCSCs in the breast cancer xenografts formed by the reinoculated BCSCs, whereas Cav-1 overexpression decreased that. The histogram (right) represents the quantitative analysis of the BCSCs proportions in three groups (left). N = 3. **(C-D)** Mitotracker-red staining assay indicated that Cav-1 overexpression elevated the mitochondrial membrane potential in the reinoculated BCSCs while Cav-1 knockdown impaired that. The histogram (right) represents the quantitative analysis of the fluorescence intensities of mitotracker-red-stained cells. Scale bar = 20 μm. N = 3. All values are presented as the mean ± SD, ^**^*P* < 0.01.

**Supplementary Figure 3. The expression levels of indicated proteins in wild type mice NBSCs, MMTV-Wnt1 mice BCSCs and non-stem MMTV-Wnt1 mice cancer cells.** (A-B) BCSCs isolated from mammary tumors of MMTV-Wnt1 mice exhibited decreased expression of Cav-1 and elevated expression of c-Myc when compared with those of NBSCs isolated from wild type mice. Scale bar = 20 μm. (C-D) Cav-1 expression levels in wild type mice NBSCs, MMTV-Wnt1 mice BCSCs and non-stem MMTV-Wnt1 mice cancer cells. The histogram (right) represents the quantitative analysis of the western blot results (left). N = 3. All values are presented as the mean ± SD, ^**^*P* < 0.01.

**Supplementary Figure 4. The correlation between Cav-1 and HIF1α expression levels as well as their prognostic value in breast cancer patients.** (**A**) Cav-1 overexpression in BCSCs led to accelerated degradation of HIF1α when the protein-synthesis inhibitor, cycloheximide (CHX, 10 ug/ml), was administrated. Meanwhile, the proteasome inhibitor, MG132 (10 μM), reversed Cav-1 overexpression-accelerated HIF1α degradation in BCSCs. N = 3. (**B**) The expression level changes of HIF1α and VHL in BCSCs after Cav-1 overexpression and knockdown. N = 3. (**C**) Representative pictures of Cav-1 expression and HIF1α expression in cancer tissues of human breast cancer patients. Scale bar = 100 μm. **(D)** Correlation analysis validated the negative correlation between Cav-1 and HIF1α expression levels in breast cancer patients. N = 83. **(E)** Joint analysis suggested that patients with Cav-1^low^/HIF1α^high^ phenotype exhibited the worst overall survival. N = 83. All values are presented as the mean ± SD, ^*^*P* < 0.05, ^**^*P* < 0.01.

**Supplementary Figure 5. Betulinic acid significantly elevated Cav-1 expression while attenuated ALDH1A1 expression in mammary tumors of MMTV-PyVT^+/−^ mice *in vivo*.** The FVB/N-Tg (MMTV-PyVT)/Nju mammary-tumor-prone mice (MMTV-PyVT^+/-^) were purchased from the Model Animal Research Center of Nanjing University (Nanjing, China). The female MMTV-PyVT^+/-^ mice (18~20 g) at 6 weeks of age were randomly divided into the control group and betulinic acid treatment group using the random number table method (n = 8 in each group), and received either saline treatment (q2d, intraperitoneally, i.p.) or betulinic acid treatment (250 mg/kg, q2d, i.p.) for 40 days. The expression levels of Cav-1 and ALDH1A1 in mammary tumor tissues were investigated by immunofluorescence assay. Scale bar = 5 μm.

**Supplementary Figure 6. The statistical analysis of the western blot results in Figure 1.** Supplementary Figure 6A applies to the western blot results in Figure 1A. Supplementary Figure 6B applies to the western blot results in Figure 1C. Supplementary Figure 6C applies to the western blot results in Figure 1E. Supplementary Figure 6D applies to the western blot results in Figure 1F. N = 3. All values are presented as the mean ± SD, ^*^*P* < 0.05, ^**^*P* < 0.01.

**Supplementary Figure 7. The statistical analysis of the western blot results in Figure 2, Figure 3 and Figure 4.** Supplementary Figure 7A applies to the western blot results in Figure 2D. Supplementary Figure 7B applies to the western blot results in Figure 3C. Supplementary Figure 7C applies to the western blot results in Figure 3I. Supplementary Figure 7D applies to the western blot results in Figure 4C. Supplementary Figure 7E applies to the western blot results in Figure 4F. Supplementary Figure 7F applies to the western blot results in Figure 4L. N = 3. All values are presented as the mean ± SD, ^**^*P* < 0.05.

**Supplementary Figure 8. The statistical analysis of the western blot results in Figure 5 and Supplementary Figure 1.** Supplementary Figure 8A applies to the western blot results in Figure 5B. Supplementary Figure 8B applies to the western blot results in Figure 5D. Figure S8C applies to the western blot results in Supplementary Figure 1C. N = 3. All values are presented as the mean ± SD, ^*^*P* < 0.05, ^**^*P* < 0.01.
