# Supplementary material for: Caveolin-1 inhibits breast cancer stem cells via c-Myc-mediated metabolic reprogramming
Source: Cell Death Dis. 2020 Jun 11;11(6):450. doi: 10.1038/s41419-020-2667-x (PMC7290025; doi:10.1038/s41419-020-2667-x)
Supplement: Supplementary file 11 — Supplementary Materials and Methods [file 41419_2020_2667_MOESM11_ESM.doc]

**Supplementary Materials and Methods**

Key materials used in this study

| REAGENT or RESOURCE SOURCE IDENTIFIER |
| --- |
| Antibodies |
| β-actin CST Cat#4970S |
| Cav-1 CST Cat#3238 |
| c-Myc CST Cat#5605 |
| LDH-A CST Cat#3582S |
| p-PDK1 CST Cat#3061 |
| CoxIV CST Cat#4850 |
| SOD2 CST Cat#13141 |
| Anti-rabbit IgG-Alexa Fluor® 555 CST Cat#4413S |
| Anti-mouse IgG-Alexa Fluor® 488 CST Cat#4408S |
| LDH-B Santa Cruz Cat#100775 |
| PGC1-α Santa Cruz Cat#13067 |
| Nrf-1 Santa Cruz Cat#13031 |
| Cav-1 Proteintech Cat#16447-1-AP |
| PDK1 Proteintech Cat#10026-1-AP |
| Myc Proteintech Cat#10828-1-AP |
| VHL Abclonal Cat#A11872 |
| RAS Abclonal Cat#A7901 |
| HIF1α Affinity Cat#AF1009 |
| ALDH1A1 Affinity Cat#BF0220 |
| CD44-FITC Thermo Fisher Cat#11-0441-85 |
| CD24-PE Thermo Fisher Cat#12-0247-42 |

Chemicals

| Dulbecco’s Modified Eagle Medium Gibco Cat#C11995500BT |
| --- |
| Fetal bovine serum Gibco Cat#10270106 |
| Penicillin and streptomycin Gibco Cat#15140122 |
| DMEM/F12 medium Gibco Cat#C11330500BT |
| Horse serum Gibco Cat#26050088 |
| B27 Gibco Cat#17504044 |
| Hydrocortisone Sigma-Aldrich Cat#07904 |
| Insulin Sigma-Aldrich Cat#I9278 |
| Bovine serum albumin Sigma-Aldrich Cat#B2064 |
| Triton X-100 Sigma-Aldrich Cat#T9284 |
| DAPI Sigma-Aldrich Cat#D9542 |
| Sodium pyruvate Sigma-Aldrich Cat#P5280 |
| Glucose Sigma-Aldrich Cat#G7528 |
| 3-bromopyruvate Sigma-Aldrich Cat#16490 |
| Human recombinant EGF STEMCELL Cat#02633 |
| Human recombinant bFGF STEMCELL Cat#78003 |
| Heparin STEMCELL Cat#07980 |

Continued

| REAGENT or RESOURCE SOURCE IDENTIFIER |
| --- |
| EpiCultTM-B medium STEMCELL Cat#05612 |
| L-glutamine Life Cat#25030081 |
| XF Base Medium Agilent Cat#102353-100 |
| X-tremegene siRNA transfection reagent Roche Diagnostics Cat#4476093001 |
| LipoFiterTM reagent Hanbio Biotechnology Cat#HB-TRLF-1000 |
| Mitotracker-red Invitrogen Cat#M22425 |

Critical Commercial Assays

| Pierce® Co-Immunoprecipitation Kit Thermo Fisher Cat#26149 |
| --- |
| XF Cell Mito Stress Test Kit Agilent Cat#103015-100 |
| XF Glycolysis Stress Test Kit Agilent Cat#103020-100 |
| ALDEFLUOR Stem Cell Identification Kit STEMCELL Cat#01700 |
| Lactate Assay Kit Sigma-Aldrich Cat#MAK064 |
| Hematoxylin and Eosin Staining Kit Beyotime Cat#C0105 |
| PrimeScript™ RT reagent Kit Takara Cat#RR047A |
| SYBR Premix Ex Taq Kit Takara Cat#RR820A |
| Ubiquitination Kit Enzo Cat#UW9920-0001 |
| Human breast cancer tissue microarray Outdo Biotech Cat#HBre-Duc140Sur-01 |

Experimental Models: Cell lines

| MCF-10A ATCC Cat#CRL-10317 |
| --- |
| MCF-7 ATCC Cat#HTB-22 |
| MDA-MB-231 ATCC Cat#HTB-26 |
| SK-BR-3 ATCC Cat#HTB-30 |
| T47D ATCC Cat#HTB-133 |
| BT-474 ATCC Cat#HTB-20 |

Experimental Models: Organisms/Strains

| Mouse: FVB.Cg-Tg(Wnt1)1Hev/J The Jackson Laboratory Stock No:002934 |
| --- |
| Mouse: Cav1^tm1Mls^/J The Jackson Laboratory Stock No: 004585 |

Plasmid and shRNA

| Recombinant plasmids for *CAV1* Vigene Biosciences Cat#CH803615 |
| --- |
| Recombinant plasmids for *RAS*  Vigene Biosciences Cat#CH891104 |
| Recombinant plasmids for *VHL* Vigene Biosciences Cat#CH800644 |
| shRNA plasmid for *CAV1* Gene Pharma Cat#A6780 |

Software

| FlowJo version 10 TreeStar https://www. flowjo.com |
| --- |
| Prism version 5 GraphPad https://www. graphpad.com |
| Gel-Pro analyzer 4 Media Cybernetics http://www.mediacy.com |
| Wave 2.6 Agilent https://www.agilent.com |
| Image J National Institutes of Health https://imagej.nih.gov/ij/ |
| SPSS 17.0 Abbott Laboratories https://www.ibm.com |

**Cell culture**

Breast cancer cell lines were maintained in Dulbecco’s Modified Eagle Medium (DMEM, Gibco, Grand Island, NY, USA) supplemented with 10% fetal bovine serum (Gibco) and 1% penicillin and streptomycin (Gibco) at 37°C in a humidified incubator containing 5% CO2. MCF-10A cells were cultured in DMEM/F12 medium supplemented with 5% horse serum, 1% penicillin and streptomycin (Gibco), 20 ng/ml recombinant human epidermal growth factor (EGF), 100 ng/ml cholera toxin, 10 μg/ml insulin (Sigma-Aldrich, Shanghai, China) and 0.5 μg/ml hydrocortisone (STEMCELL Technologies, Vancouver, Canada).

**Western blotting and immunoprecipitation**

The protein lysates were applied to SDS-PAGE, transferred to a polyvinylidene fluoride microporous membrane (Millipore, Billerica, MA, USA) and probed with primary antibodies for β-actin (no.4970S), Cav-1 (no.3238S), c-Myc (no.5605), LDH-A (no.3582S), p-PDK1 (no.3061S), CoxIV (no.4850), SOD2 (no.13141) (Cell Signaling Technology, CST, Boston, MA, USA), Nrf-1 (sc-13031), LDH-B (sc-100775), PGC1-α (sc-13067) (Santa Cruz, CA, USA), Cav-1 (no.16447-1-AP), PDK1 (no.10026-1-AP) (Proteintech, Chicago, USA), VHL (A11872, Abclonal, Wuhan, China), RAS (A7901, Abclonal) or HIF1α (AF1009, Affinity, Changzhou, China). Immunoprecipitation assay was conducted by using the Pierce® Co-Immunoprecipitation Kit (no.26149, Thermo Fisher, Hudson, NH, USA) according to the manufacturer’s instructions. Primary antibodies for VHL (A11872, Abclonal), c-Myc (no.10828-1-AP, Proteintech) and Cav-1 (no.16447-1-AP, Proteintech) were used in this assay. The relative expression levels of proteins between groups were calculated by comparing optical densities of bands using the Gel-Pro analyzer 4 software (Media Cybernetics, USA).

**Transmission electron microscopy observation**

The caveolae structures in different cells were observed and digitally photographed by the JEM-1400 transmission electron microscope (JEOL, Tokyo, Japan) as described previously[^1^](#_ENREF_1).

**Oxygen consumption rate (OCRs) and extracellular acidification rate (ECARs)**

The Seahorse XF24 extracellular flux analyzer (Seahorse Bioscience) was used to detect OCRs to measure mitochondrial respiration, and ECARs to determine glycolysis. Briefly, cells were seeded into XF24 cell culture microplates at a density of 3~10×10^4^ cells per well (4×10^4^ for human breast cancer cells and the reinoculated BCSCs; 10×10^4^ for MCF-10A cells; 3×10^4^ for mouse NBSCs and mouse BCSCs), and cultured overnight. Meanwhile, the XF24 cartridge was equilibrated with the calibration solution overnight at 37℃. For measuring OCRs, XF assay medium (10 mM glucose, 2 mM glutamine and 1 mM sodium pyruvate in XF base medium prepared and pH-adjusted to 7.4 on the day of the experiment) was used to prepare the cellular stress-inducing reagents, 1 μM oligomycin, 1μM carbonyl cyanide 4-(trifluoromethoxy)phenylhydrazone (FCCP), 0.5 μM antimycin A and 0.5 μM rotenone (final concentration). For ECARs, the XF assay media (1 mM glutamine in XF base medium, pH 7.4) was used to prepare glycolysis stress reagents including 10 mM glucose, 1 μM oligomycin and 50 mM 2-DG. All the reagents were loaded in the ports according to the manufacturer’s instructions. After OCRs and ECARs detection, cells in each well of the cell culture microplates were collected and counted using a cell counting chamber. The relative cell numbers were calculated and used to normalize the OCRs and ECARs values. The cell energy phenotype profiles of indicated cells were obtained by analyzing the OCRs and ECARs values.

**Colony formation assay**

For colony formation assay, cells were seeded in a 6-well plate at a density of 500 cells per well. After attachment, cells were treated as indicated and then continued to culture for 2 weeks. The resultant colonies were fixed with 4% paraformaldehyde and then stained with coomassie blue.

**Transfection of plasmid and siRNA**

The commercialized recombinant plasmids for *CAV1* (no.CH803615), *RAS* (no.CH891104) and *VHL* (no.CH800644) as well as the non-target control plasmids were purchased from Vigene Biosciences (Jinan, China). The shRNA plasmid for *CAV1* (no.A6780) was obtained from Gene Pharma Co, LTD (Shanghai, China). All the above plasmids were transfected into indicated cells using LipoFiter^TM^ reagent (Hanbio Biotechnology Co, LTD. Shanghai, China) according to the manufacturer’s protocol. The commercialized siRNAs targeting *CAV1* or *VHL* as well as the scramble siRNAs were also purchased from Vigene Biosciences and transfected into indicated cells using X-tremegene siRNA transfection reagent (Roche Diagnostics, Shanghai, China).

**Mitotracker-red staining**

Cells were stained with 100 nM mitotracker-red solution (M22425, Invitrogen, Shanghai, China) for 30 minutes. Subsequently, cells were washed with PBS and observed under the BX53 fluorescent microscope (Olympus, Center Valley, PA, USA). The fluorescence intensities of images were quantified by the Image J software (National Institutes of Health).

**Stem cell population analysis, sorting and mammosphere formation assay**

Human breast CSCs population analysis and sorting were conducted as we have previously reported[^2^](#_ENREF_2). Briefly, human breast cancer cells were incubated with CD44-fluorescein isothiocyanate (FITC) and CD24-PE antibodies (Thermo Fisher, Hudson, NH, USA) at 4 °C for 30 minutes. After incubation, cells were washed once with PBS and subjected to analysis using a FACSAria III flow cytometer (BD Biosciences). The CD44^+^/CD24^−/low^ subpopulation was quantified, collected and defined as human breast CSCs. Hyperactive aldehyde dehydrogenase (ALDH) activity is closely related to the physiological properties of stem cells[^3^](#_ENREF_3). In the present study, ALDH staining assay was conducted to discriminate mice stem cells from their non-stem counterparts. Briefly, mice mammary epithelial cells and mice primary cancer cells were isolated from mice mammary tissues or mice mammary tumors respectively by mechanical and enzyme digestive methods[^4^](#_ENREF_4). Then, mice mammary epithelial cells and mice primary cancer cells were incubated with ALDEFLUOR™ (STEMCELL) at 37 °C for 30 minutes to mark ALDH^+^ cells. After incubation, cells were washed once with PBS and subjected to analysis using a flow cytometer (Beckman Coulter, Fullerton, CA, USA). The ALDH^+^ subpopulation from mice mammary epithelial cells was collected and defined as mice NBSCs while the ALDH^+^ subpopulation from mice primary cancer cells was collected and defined as mice BCSCs. DEAB denotes diethylaminobenzaldehyde. DEAB a specific inhibitor of ALDH activity, and is used to control for background fluorescence in the ALDH staining assay. In addition, mice stem cell population analysis was conducted by flow cytometry using the ALDEFLUOR Stem Cell Identification Kit (no.01700, STEMCELL). To ensure that the sorted BCSCs subpopulations were reliable, both the human BCSCs and mice BCSCs were subjected to a tumorigenesis assay as we have previously reported [^2^](#_ENREF_2) and only these cells which successfully formed tumors in mice were used in further assays. In terms of mice BCSCs, the sorted mice BCSCs were divided into multiple cell culture flasks and cultured in stem cell culture medium continually. To ensure that the sorted mice BCSCs subpopulations were reliable, the sorted mice BCSCs in one cell culture flask were harvested and subjected to a tumorigenesis assay as we have previously reported[^2^](#_ENREF_2). The remaining mice BCSCs which have the same source with the tumorigenic mice BCSCs were used in further assays. In terms of human BCSCs, as they were sorted from cultured breast cancer cells *in vitro*, and not “educated” *in vivo*, all the human BCSCs sorted from cultured breast cancer cells *in vitro* were resuspended in matrigel and reinoculated into the mammary fat pads of mice. Two weeks later, the reinoculated BCSCs were isolated from successfully-formed tumor xenografts by mechanical and enzyme digestive methods and sorted again before they were subjected to further studies. For mammosphere formation assay, the sorted BCSCs or NBSCs were cultured in ultralow attachment plates in DMEM/F12 medium supplemented with 1% penicillin-streptomycin (Gibco), 2% B27 supplement (Gibco), 20 ng/ml EGF, 5 μg/ml insulin and 0.4% bovine serum albumin (Sigma). The number and size of mammospheres were quantified microscopically.

**Lactate concentration detection assay**

The effect of Cav-1 on lactate production in breast CSCs was investigated. Briefly, 1×10^6^ cells were suspended in 150 μl cold PBS solution and then lysed by the VCX105 ultrasonic crushing equipment (SONICS, Shanghai, China). Subsequently, the lactate levels in cell lysates were measured using a Lactate Assay Kit (no.MAK064, Sigma) according to the manufacturer’s instructions.

**Mammary whole mounting, HE and Immunohistochemistry**

Mammary tissues were collected from female mice and then stretched on the glass slides. Slides were incubated in Metha-Carnoy’s Fixative for 12 hours and then stained with Carmine Alum overnight. When the Carmine had penetrated the entire tissue, the slides were incubated in 100% ethanol for 2 hours and then in xylene for 6 hours. Lastly, samples were digitally photographed using a MZ7.5 stereomicroscope (Leica, Wetzlar, Germany). HE staining was carried out using the Hematoxylin and Eosin Staining Kit (C0105, Beyotime, Nanjing, China) according to the manufacturer’s instructions. Immunohistochemistry was conducted as we have reported previously[^2^](#_ENREF_2). Samples were stained with Cav-1, c-Myc or VHL, and digital images of stained sections were captured using the BX53 upright metallurgical microscope (Olympus).

**Isolation and three**-**dimensional culture of mice mammary epithelial cells**

The isolation of mice mammary epithelial cells was carried out according to the monolayer culture of mice mammary epithelial cells technical bulletin provide by the STEMCELL Technologies Ltd[^4^](#_ENREF_4). The isolated mice mammary epithelial cells were cultured in EpiCult^TM^-B medium (no.05612) supplemented with 10 ng/ml human recombinant EGF (no.02633), 10 ng/ml human recombinant bFGF (no.78003) and 4 μg/ml heparin (no.07980) (STEMCELL). The three-dimensional culture of mice mammary epithelial cells was conducted as described previously[^4^](#_ENREF_4).

**Immunofluorescence**

For immunofluorescence analysis, cells were fixed with 4% paraformaldehyde for 20 mins, washed three times with PBS and then permeabilized with 0.25% Triton X-100 for 20 mins. After blocking in 5% BSA at room temperature for 1 h, cells were incubated with the Cav-1 antibody, c-Myc antibody (CST) or the ALDH1A1 antibody (Affinity) overnight at 4℃, followed by an incubation with the corresponding secondary antibodies including Alexa Fluor® 555 conjugated-anti-rabbit IgG (no.4413S, CST) or Alexa Fluor® 488 conjugated-anti-mouse IgG (no.4408S, CST). 4', 6-diamidino-2-phenylindole (DAPI, Sigma) was used to visualize the nuclei. Fluorescence images were obtained using a LSM710 confocal microscope (Zeiss, Jena, Germany).

**QPCR**

Total RNA was extracted with Trizol and reverse transcribed to complementary cDNA using the PrimeScript™ RT reagent Kit (Takara, Shiga, Japan) in accordance with the manufacturer’s instructions. RT-PCR was performed using the SYBR Premix Ex Taq Kit (Takara) and the ABI Prism 7500 Sequence Detector System (Applied Biosystems, Foster City, CA, USA). Primer sequences of *MYC* were 5′-GCTGCTTAGACGCTGGATTT-3′ (forward) and 5′-TAACGTTGAGGGGCATCG-3′ (reverse). Primer sequences of *β-ACTIN* were 5′- CCAACCGCGAGAAGATGA-3′ (forward) and 5′- CCAGAGGCGTACAGGGATAG -3′ (reverse).

***In vitro* ubiquitination assay**

The ubiquitination of c-Myc was investigated using the Ubiquitination Kit (UW9920-0001, Enzo, NY, USA) according to the protocol with some changes. Briefly, the reaction was carried out at 37℃ in a 60 μl mixture containing 20 U/ml inorganic pyrophosphatase, 5 mM dithiothreitol, 5 mM Mg-ATP, 100 nM E1, 2.5 mM E2 (UbcH5b), 120 μg total protein extracts and 2.5 mM biotin-labeled ubiquitin. After incubated for 5 hours, the reaction was quenched by addition of 60 μl non-reducing gel-loading buffer and separated using 12% SDS-polyacrylamide gel electrophoresis. To get accurate results, the PAGE gel was run for a relatively longer time until protein bands smaller than 55 kDa ran out of the bottom line of the gel as judged by the protein molecular weight marker. Then the protein was transferred to a polyvinylidene difluoride membrane and probed by c-Myc antibody (10828-1-AP, Proteintech) to detect ubiquitination level of c-Myc.

**Bioinformatics analysis**

For Cancer Genome Atlas (TCGA) analysis, the cBio Cancer Genomics Portal (http://cbioportal.org) was applied to analyze and visualize the mRNA alternation(s) of *CAV1* and *MYC*. The case set was defined as 2509 sequenced breast cancer patients in METABRIC, Nature 2012 & Nat Commun 2016[^7^](#_ENREF_7). Patients were classified into two risk groups based on mRNA alternations and censored for overall survival. All TCGA data included in this manuscript are in compliance with the TCGA Ethics, Law, and Policy Group, which are in compliance with the Helsinki Declaration. For *CAV1* gene-expression analysis in BCSCs and NBSCs, the GSE6883 dataset [^8^](#_ENREF_8) on the platform of GPL96 [HG-U133A] Affymetrix Human Genome U133A Array was retrieved from the National Center for Biotechnology Information (NCBI) Gene Expression Omnibus (GEO) database (<http://www.ncbi.nlm.nih.gov/geo>) and then analyzed by Gene-Cloud Biotechnology Information (GCBI, https://www.gcbi.com.cn/gclib/html/index).

**References**

1. Mondal, S. *et al.* HSulf-1 deficiency dictates a metabolic reprograming of glycolysis and TCA cycle in ovarian cancer. *Oncotarget* **6**, 33705-33719 (2015).

2. Wang, Z. *et al.* Caveolin-1 mediates chemoresistance in breast cancer stem cells via beta-catenin/ABCG2 signaling pathway. *Carcinogenesis* **35**, 2346-2356 (2014).

3. Wang, Y.P. & Lei, Q.Y. Perspectives of Reprogramming Breast Cancer Metabolism. *Advances in experimental medicine and biology* **1026**, 217-232 (2017).

4. Linnemann, J.R. *et al.* Quantification of regenerative potential in primary human mammary epithelial cells. *Development* **142**, 3239-3251 (2015).

5. Cerami, E. *et al.* The cBio cancer genomics portal: an open platform for exploring multidimensional cancer genomics data. *Cancer discovery* **2**, 401-404 (2012).

6. Gao, J. *et al.* Integrative analysis of complex cancer genomics and clinical profiles using the cBioPortal. *Science signaling* **6**, pl1 (2013).

7. Pereira, B. *et al.* The somatic mutation profiles of 2,433 breast cancers refines their genomic and transcriptomic landscapes. *Nature communications* **7**, 11479 (2016).

8. Liu, R. *et al.* The prognostic role of a gene signature from tumorigenic breast-cancer cells. *The New England journal of medicine* **356**, 217-226 (2007).
